# Extraction, Purification, and Characterization of Olive (*Olea europaea* L., cv. Chemlal) Polyphenol Oxidase

**DOI:** 10.1021/acs.jafc.3c07776

**Published:** 2024-01-30

**Authors:** Ala eddine Derardja, Matthias Pretzler, Malika Barkat, Annette Rompel

**Affiliations:** † Universität Wien, Fakultät für Chemie, Institut für Biophysikalische Chemie, Josef-Holaubek-Platz 2, 1090 Wien, Austria; ‡ Laboratoire Bioqual, INATAA, Université Frères Mentouri, Constantine 1, Route de Ain El-Bey, 25000 Constantine, Algeria

**Keywords:** polyphenol oxidase, olive, Chemlal, purification, characterization, phenolics

## Abstract

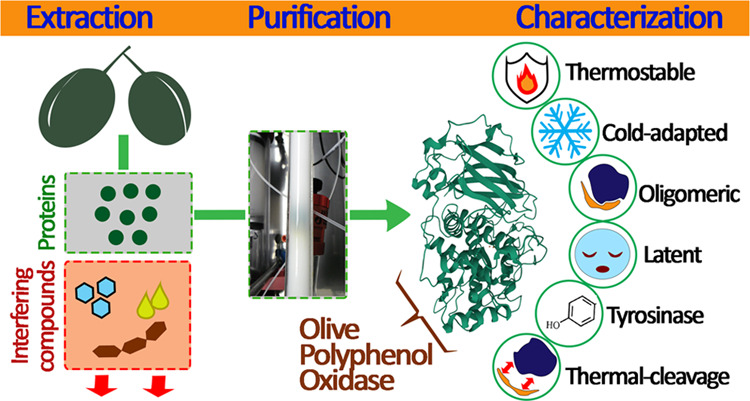

Among fruits susceptible to enzymatic browning, olive polyphenol oxidase (*Oe*PPO) stood out as being unisolated from a natural source until this study, wherein we successfully purified and characterized the enzyme. Sodium dodecyl sulfate-polyacrylamide gel electrophoresis (SDS-PAGE) of heated and nonheated *Oe*PPO revealed distinct molecular weights of 35 and 54 kDa, respectively, indicative of its oligomeric nature comprising active and C-terminal subunits. *Oe*PPO displayed latency, fully activating with 5 mM SDS under optimal conditions of pH 7.5 and 15 °C. The enzyme demonstrated monophenolase activity and showcased the highest efficiency toward hydroxytyrosol. Despite its low optimal temperature, *Oe*PPO exhibited high thermal resistance, maintaining stability up to 90 °C. However, beyond this threshold, the oligomeric enzyme disassociated, yielding a denatured main subunit and C-terminal fragments. Six *Oe*PPO genes were found in the fruits. Tryptic digestion identified the enzyme as mature *Oe*PPO1 (INSDC OY733096), while mass spectrometry detected the active form mass alongside several C-terminal fragments, revealing potential cleavage sites (Gly407, Tyr408).

## Introduction

Olive (*Olea europaea L.*), a significant fruit crop, finds widespread cultivation in the Mediterranean basin and other global regions.^1^ This fruit is commonly utilized for producing olive oil or for direct consumption as table olives. Renowned for their health benefits, olives and olive oil feature a substantial content of bioactive compounds, predominantly antioxidants.^1^ Among these compounds, phenolic compounds stand out as primary antioxidants.^1^ However, akin to many other fruits rich in phenolic compounds, olives are notably susceptible to enzymatic browning, a reaction triggered when the fruit is cut, bruised, or mechanically processed.^2^ Enzymatic browning constitutes a natural process prevalent in fruits and vegetables, instigated by enzymes that catalyze a cascade of chemical reactions commencing with phenolic oxidation and culminating in browning.^3,4^ This enzymatic oxidation is mainly triggered by polyphenol oxidase (PPO), a type-III copper-containing oxidoreductase present in various organisms, encompassing plants, animals, fungi, and bacteria,^5^ characterized by a type-III copper center comprising two copper ions, each coordinated by three conserved histidine residues.^4−6^ PPOs play a pivotal role in initiating browning reactions. Specifically, they catalyze the ortho-hydroxylation of monophenols to catechols (monophenolase activity, EC 1.14.18.1) and the ensuing oxidation of catechols to corresponding *o*-quinones (diphenolase activity, EC 1.10.3.1).^4,5^ The resultant compounds exhibit heightened reactivity and can spontaneously polymerize, giving rise to a diverse spectrum of colored products, including brown-hued melanins.^3,5^ These products exert adverse effects on the sensory attributes of olives, potentially disrupting their flavor, aroma, and texture.^7^ Furthermore, the presence of these pigmented byproducts detracts from the visual appeal of olives, thus diminishing their desirability among consumers.^3^ The oxidation process also leads to a significant reduction in the concentration of phenolic compounds within olives and olive-derived products.^2^

Most plant PPOs are reported to exist in a latent form.^4,5,8,9^ The latency of PPOs can be overcome through several methods, including the addition of detergents, alcohols, acids, and alkalis, mild heat treatment, treatment with proteases, and sonication.^4^ Among these activating agents, the detergent sodium lauryl sulfate (SDS) is highly effective in detecting and activating latent PPOs.^4,10^ SDS induces conformational changes in the N- and C-termini of the latent form, resulting in the exposure of the active site and facilitating substrate catalysis.^10^

Due to their significant role in causing undesirable browning of fruits and vegetables, plant PPOs have undergone extensive research over the past decades.^4,5,8^ These studies have delved into the enzyme’s molecular and biochemical properties, aiming to gain better control over its detrimental effects. Consequently, many of the most commercially relevant plant PPOs have been isolated from natural sources, subjected to purification, and thoroughly characterized.^4,8,9^ Extensive investigations were carried out on PPOs from the most affected common fruits such as apple, grape, apricot, and banana, encompassing numerous cultivars.^3−5,9^ The comprehensive scrutiny of PPO from diverse sources and cultivars is necessitated by the molecular and biochemical variations observed among PPOs isolated from distinct origins.^4,5,8^

In contrast, and despite its pivotal role in defining the quality of olives and olive oil, olive polyphenol oxidase (*Oe*PPO) has remained unexplored in terms of native proteins isolated from natural sources. The sole study was focused on the identification and characterization of olive PPO genes and applied recombinant *Oe*PPO.^11^ Out of four recombinant *Oe*PPOs produced in this study,^11^ only one (*Oe*PPO3) exhibited monophenolase activity, while another one (*Oe*PPO4) was devoid of all activity. Thus, except for this recent study, all prior reports have been based on crude extracts.^2,7,12^ These reports showed pronounced changes in PPO activity during ripening,^7,12^ between olive cultivars^2^ and tissues^2,12^ of olive trees. The limited documentation available on pure *Oe*PPO and its characterization does not stem from the lack of recognition of its importance; rather, it arises from the formidable challenges encountered during the extraction and purification of PPO from olive fruits. These challenges primarily arise due to the presence of multiple interfering compounds, including olive oil, pectins, phenolic compounds, and pigments.^13^ The intricate interplay of these compounds complicates the extraction of a purifiable enzyme solution, posing a significant obstacle to overcome.

Therefore, in this study, we present a comprehensive protocol for the extraction of *Oe*PPO that effectively eliminates the majority of interfering compounds. Subsequently, we employ chromatographic purification to isolate the enzyme obtained from the natural source, and more importantly, we explore herein both the biochemical and molecular properties of the purified *Oe*PPO. Additionally, *Oe*PPO coding genes of the Chemlal cultivar were identified and served as the basis for the molecular characterization of the purified enzyme. The outcomes of this study are poised to provide valuable insights, enhancing our comprehension of enzymatic browning reactions in olives and offering refined avenues for control.

## Materials and Methods

### Plant Material

Mature (full ripeness) olives (*Olea europaea* L., cv. Chemlal) were bought from a local garden in Bordj Bou Arreridj, Algeria, in November 2022. Within 24 h of harvesting, the fruits were transported to the laboratory at the University of Vienna and stored at −80 °C until they were utilized as a source of PPO or DNA.

### Protein Extraction and Removal of Interfering Compounds

An optimized two-step approach was utilized to extract olive PPO while minimizing the presence of interfering compounds. The process involved acetone precipitation, followed by aqueous two-phase separation (ATPS). Initially, frozen olives were deseeded, promptly shock-frozen in liquid nitrogen, and then ground using a mortar and pestle. Two hundred and fifty grams of the resulting frozen olive powder was homogenized in 1 L of cold acetone (−25 °C) for 2 min. The slurry was then filtered through filter paper, and the residue was subjected to four additional extractions with 300 mL of cold acetone each.

The resulting acetone paste was air-dried at room temperature until all of the traces of acetone had evaporated. To further eliminate any remaining interfering compounds, an ATPS procedure was employed, following the protocol outlined by Molitor et al.^14^ with some modifications. Notably, 11 g of acetone powder was homogenized with 400 mL of 100 mM sodium phosphate buffer (pH 6.8), containing 4% (v/v) Triton X-114, 0.5% (w/v) sodium ascorbate, 1% (w/v) polyvinylpolypyrrolidone (PVPP), 1 mM phenylmethylsulfonyl fluoride (PMSF), and 2 mM benzamidine hydrochloride, using an Ultra-Turrax homogenizer. The resulting homogenate was stirred for 30 min at 4 °C and then subjected to centrifugation at 16,000*g* for 20 min at 4 °C. The supernatant was filtered through cheesecloth, and 15 g/L of ammonium sulfate were added, inducing liquid–liquid phase separation upon warming to 8–12 °C. After centrifugation at 16,000*g* for 10 min at 12 °C, the detergent-rich bottom phase was discarded, while the preserved upper phase was enriched with Triton X-114 to achieve an approximate final concentration of 4% (v/v). The cloudy solution was stirred for 15 min at 8–12 °C and subsequently centrifuged at 16,000*g* for 10 min at 12 °C. The lower phase was once more discarded, and ammonium sulfate was added to the upper phase until it reached a final concentration of 30% saturation. The suspension was then cooled to 4 °C and left for 45 min. Following centrifugation (16,000*g* for 45 min at 4 °C) and filtration, PEG-4000 was added to achieve a final concentration of 4.5% (w/v). The resulting turbid solution underwent another round of centrifugation at 16,000*g* for 10 min at 4 °C. The detergent-rich top phase was discarded, while the lower phase was subjected to another cycle of ATPS. This procedure was repeated twice with decreasing amounts of PEG-4000 (4 and 3.5% w/v, respectively).Ammonium sulfate was added to the resulting clear enzyme solution until it reached an approximate final concentration of 85% saturation, and then the mixture was stored at 4 °C overnight. The precipitated fraction was separated from the supernatant by centrifugation at 30,000*g* for 30 min at 4 °C. To obtain the crude extract, the precipitate was dissolved in 250 mL of 10 mM Tris–HCl buffer (pH 8.25) supplemented with 10 mM sodium ascorbate.

### Purification by Fast Protein Liquid Chromatography

**Figure 1:**
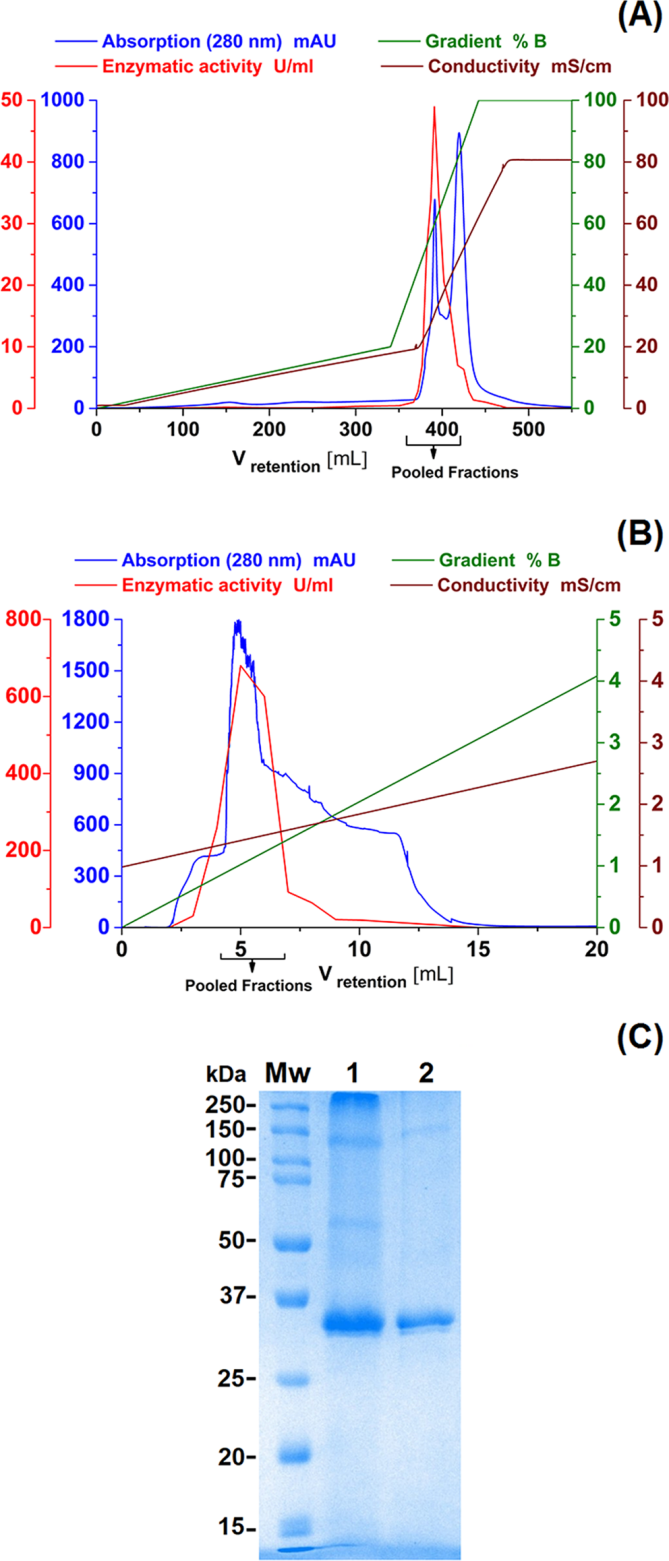
Chromatographic separation steps. (A) AEX chromatography using Q-Sepharose. (B) CEX chromatography using Mono S. Legend: (blue) UV absorbance at 280 nm (mAU), (red) *Oe*PPO activity (U/mL), (green) elution gradient (% buffer B with 1 M NaCl), and (brown) conductivity (mS/cm). (C) Denaturing SDS-polyacrylamide gel electrophoresis (PAGE) of *Oe*PPO during purification steps: (Mw) molecular weight marker (values are given in kDa), (1) pooled fractions after anion exchange on Q-Sepharose and (2) cation exchange on Mono S.

The olive PPO extract was dialyzed (through dialysis tubing with a molecular weight cutoff of 14 kDa) at 4 °C against 10 mM Tris–HCl buffer (pH 8.25) containing 10 mM sodium ascorbate. This dialysis process involved two buffer changes, with each change lasting for 3 h. Subsequently, a second round of dialysis was performed overnight at 4 °C against three changes of Tris–HCl buffer (10 mM, pH 8.25). After dialysis, the suspension underwent centrifugation at 30,000*g* for 45 min at 4 °C. It was then filtered through a 0.45 μm polyethersulfone membrane before being processed using an ÄKTA fast protein liquid chromatography system (FPLC) placed in a refrigerator at 4 °C. Throughout the purification process, the fractions were continuously monitored for both protein content (UV-absorption at 280 nm) and enzymatic activity (diphenolase activity) using catechol as the substrate in 50 mM sodium phosphate buffer at pH 6.8, containing 2 mM SDS as an activator. The protein solution was loaded onto an anion-exchange (AEX) column (Q-Sepharose FF, 20 mL) pre-equilibrated with 10 mM Tris–HCl, pH 8.25, solution. Bound proteins were eluted with a linear gradient of sodium chloride (0–200 mM in 340 mL, 200–1000 mM in 100 mL) at a flow rate of 2 mL/min (Figure 1A). Fractions (53 mL) showing enzymatic activity were pooled and concentrated through ultrafiltration (using a 30 kDa molecular weight cutoff) via centrifugal force at 4000*g* and 4 °C. The concentrated protein solution was subjected to a cation exchange (CEX) chromatography (Figure 1B) using a Mono S HR 5/50 GL column (1 mL). The column was pre-equilibrated with 20 mM sodium acetate buffer at pH 5.2, and elution was performed with a linear gradient of sodium chloride (0–300 mM in 160 mL, 300–1000 mM in 100 mL) at a flow rate of 0.5 mL/min. Fractions (3 mL) containing catecholase activity were pooled, washed with 10 mM Tris–HCl buffer at pH 8.0, and concentrated using ultrafiltration. The purified enzyme was stored at 4 °C in Tris–HCl buffer (10 mM, pH 8.0). Tris–HCl buffer at pH 8.0 was identified as being suitable for *Oe*PPO storage, preventing activation at low pH and enzyme precipitation.

### Enzyme Activity and Protein Concentration

*Oe*PPO activity measurements were carried out spectrophotometrically at 25 °C using a 96-well microplate reader (Infinite M200, Tecan). The assay mixture, with a volume of 200 μL, was monitored for the increase in absorbance at 410 nm as the enzyme converted catechol, starting from ∼25 s after mixing (time required for pipetting and initiating the microplate reader) and continuing until the reaction curve reached a plateau phase (after ∼15 min). The standard reaction mixture consisted of 0.5 μg of enzyme and 10 mM catechol in 50 mM sodium phosphate buffer (pH 7.5) along with the optimal concentration of SDS (5 mM) as determined. The exclusion of SDS significantly weakened *Oe*PPO activity and did not yield usable absorption–time curves. *Oe*PPO activity was assessed by calculating the slope of the initial linear segment of the experimental curves, where the absorbance was plotted against time. The activity was expressed in U/mg, with one unit of enzymatic activity (U) defined as the amount of enzyme that catalyzed the formation of 1 μmol of quinones per minute (1 U = 1 μmol/min). All experiments were conducted in triplicate, and the protein concentrations in the enzyme solutions were determined using the Bradford method^15^ with bovine serum albumin as a standard.

### Gel Electrophoresis

Denaturing and partially denaturing sodium dodecyl sulfate-polyacrylamide gel electrophoresis (SDS-PAGE) were conducted following the method described by Laemmli^16^ using a Mini-PROTEAN Tetra Cell from Bio-Rad. The heated samples were incubated at 99 °C for 5 min using a Thermomixer comfort system from Eppendorf. For reduced samples 350 mM 2-mercaptoethanol were added to the sample buffer (75 mM Tris−HCl pH 6.8, 70 mM SDS, 10% (v/v) glycerol, 0.5 g/L bromophenol blue) prior to the heating step. Subsequently, the samples (heated or nonheated, reduced or nonreduced) were applied to 12% polyacrylamide gels and run at 120 V. After running, gels were dyed with Coomassie Brilliant Blue G-250 to visualize the protein bands. Molecular weights were estimated by comparing the migration of target proteins with molecular weight markers (Precision Plus Protein Standard Dual Color, Bio-Rad). For the activity gel of *Oe*PPO, a 12% SDS-PAGE was conducted using nonreduced and nonheated samples. The staining of *Oe*PPO was carried out by immersing the gel in 50 mM sodium phosphate buffer (pH 7.5) containing 0.1 M catechol at 25 °C. After incubation for 30 min on an orbital shaker, the gel was carefully washed with distilled water and then photographed against a white background.

### Copper Ion Content Determination

The method described by Hanna et al.^17^ was employed to quantify the copper ion content of the purified *Oe*PPO. Thus, 600 μg of purified enzyme was mixed with 50 mM sodium ascorbate, and then the total volume was brought to 400 μL using 100 mM sodium phosphate buffer with a pH of 6.0. Subsequently, 600 μL of a 0.5 g/L 2,2′-biquinoline solution in glacial acetic acid was added. After 10 min of incubation, the absorbance was measured at 546 nm (ε = 6300 M^–1^ cm^–1^). The copper ion content in the samples was ascertained for blank corrected samples.

### Effect of SDS

The effect of different concentrations of SDS (0–100 mM) on *Oe*PPO activity was investigated. The enzyme activity was determined following the previously described method and expressed as a percentage of the residual activity relative to the SDS concentration that exhibited optimal activity set to 100%.

### Effect of pH

To ascertain the pH conditions that yield optimal *Oe*PPO activity, an extensive range of 50 mM buffers was employed, comprising sodium citrate (pH 1.5–7.5), sodium phosphate (pH 6.0–7.5), and Tris–HCl (pH 7.0–9.0), serving as the reaction buffer. The evaluation of *Oe*PPO activity as a function of pH was performed in the presence and absence of SDS using the same assay conditions as those previously described. The quantification of *Oe*PPO activity was expressed as the percentage of residual activity at the pH that exhibited the peak enzyme performance. This optimal pH value was subsequently adopted for all ensuing studies.

### Effect of Temperature

The optimal temperature for *Oe*PPO activity was determined over a range of 5–45 °C in 800 μL of assay mixture, comprising 2 μg of enzyme and 10 mM catechol in 50 mM sodium phosphate buffer (pH 7.5). The experiments were assessed using a Shimadzu UV spectrophotometer (UV-1800) linked to a thermostat (Julabo F25) via a circulating water bath, ensuring precise control and maintenance of the desired temperature. Before the addition of the enzyme solution, the substrate and buffer were incubated for 10 min in the same water bath (Julabo F25) at the designated temperature. *Oe*PPO activity was expressed as a percentage of residual activity compared to the enzymatic activity at optimal temperature (100%).

### Thermal Stability

Heat treatments of *Oe*PPO were carried out at 70, 80, 90, and 100 °C for varying periods (1, 5, 10, 20, and 30 min) in a temperature-controlled water bath. The selected temperature range (70–100 °C) was established based on an extended preliminary temperature range (data not shown). *Oe*PPO (0.5 μg) was incubated in 50 mM sodium phosphate buffer (pH 7.5) at the desired temperature and for the specified period, followed by rapid cooling in an ice bath for 10 min and subsequent warming up to 25 °C. The residual enzyme activity was analyzed with 10 mM of catechol in 200 μL of assay mixture, and the obtained results were expressed as a percentage of residual activity relative to the untreated enzyme (100%).

To better investigate the thermal stability of our enzyme, a thermal shift assay^18,19^ was conducted to measure the melting point of the purified *Oe*PPO. The assay was performed in triplicate using polymerase chain reaction (PCR) tubes (Axygen, Inc., Corning) in a real-time PCR instrument (Mastercycler ep-realplex, Eppendorf). The reaction solutions contained 10 μM enzyme and 4 × SYPRO Orange (Sigma-Aldrich) in 50 mM sodium phosphate buffer (pH 7.5). The samples were gradually heated with an increment of 0.5 K per 30 s from 4 to 99 °C in the PCR machine. During this process, the fluorescence intensity was meticulously monitored at a wavelength of 550 nm, with excitation occurring at 470 nm. Subsequently, the acquired fluorescence intensity data points for each enzyme-containing solution were utilized to construct plots correlating the fluorescence intensity with temperature for thermal stability analysis.

A denaturing SDS-PAGE analysis was performed as described previously with the sole modification during the heating step by testing four distinct temperature conditions: 70, 80, 90, and 100 °C for 5 min each. The samples were loaded onto a single gel and stained with Coomassie Blue. The obtained results were compared to those derived from the two aforementioned prior experiments.

### Enzyme Kinetics and Substrate Specificity

*Oe*PPO activity was assayed as explained above using monophenols (tyramine, tyrosol) and diphenols (hydroxytyrosol, oleuropein, dopamine, catechol, 4-methylcatechol, and 4-*tert*-butylcatechol) (Figure S1), at various molarities. The *K*_m_ value and maximum velocity *V*_max_ were determined by nonlinear regression. The maximal turnover rate (*k*_cat_) was calculated by dividing the total substrate converted per second by the total molecules of *Oe*PPO in the reaction mixture. The molar absorption coefficients (ε_max_) of the formed quinones in 50 mM sodium phosphate buffer at pH 7.5 were evaluated using a quantitative oxidation method, wherein small quantities of the respective diphenols were oxidized by an excess of sodium periodate (NaIO_4_), following the procedure described by Muñoz et al.^20^

### Effect of Metal Ions on Enzymatic Activity

The effects of a wide range of metal ion salts (AlCl_3_, CaCl_2_, CoCl_2_, CrCl_3_, CuSO_4_, FeCl_2_, KCl, MgCl_2_, MnCl_2_, NaCl, NiSO_4_, and ZnCl_2_) on *Oe*PPO activity was investigated for different molarities (1, 5, 10, and 100 mM). The activity was determined as described above in 50 mM sodium citrate buffer containing 5 mM SDS, and the results were expressed as a percentage of residual activity relative to the control mixture (100%) without metal ions.

### Effect of Inhibitors

Various inhibitors (ascorbic acid, citric acid, reduced glutathione, histidine, kojic acid, l-cysteine, *N*-phenylthiourea, sodium metabisulfite, and succinic acid) were evaluated for their effectiveness as inhibitors of *Oe*PPO activity at multiple concentrations. The optimum concentration range for each inhibitor was established through preliminary experiments (data not shown). *Oe*PPO activity was measured using the same assay conditions previously described, and the results were expressed as the percentage of residual activity relative to the control mixture (100%) without inhibitors. The half inhibitory concentration (IC_50_) was determined for each inhibitor using nonlinear regression.

### Protein Identification

The band at 55 kDa obtained by SDS-PAGE was isolated from the gels and subjected to tryptic digestions, then analyzed by nano UHPLC-ESI-MS/MS using a high-resolution Orbitrap mass spectrometer (Dionex Ultimate 3000 RSLCnano, Q Exactive Orbitrap, Thermo Scientific). The data analysis was performed using Proteome Discoverer 1.4, with a search conducted against the sequences of the genes cloned in this study (see below; Figure S2), as well as the *O. europaea* entries in the UniProt database. Peptide mass tolerance was set at 5 ppm, and the fragment mass tolerance was set at 0.5 Da. Variable modifications allowed for the oxidation of methionines and carbamidomethylation of cysteines.

### DNA Isolation and Cloning

DNA was isolated from half the flesh and peel of a single ripe olive (≈700 mg wet weight) using an SDS-based isolation procedure developed for olive fruits based on methods published by Turci et al.^21^ and Bi et al.^22^ SDS, polyvinylpyrrolidone (PVP), and activated charcoal (see buffer recipe below) were added to the mortar and ground along with the frozen fruit under liquid nitrogen. The frozen powder was transferred to a 2 mL reaction vial filled with 1 mL of extraction buffer (100 mM Tris–HCl pH 8, 50 mM Na_3_EDTA pH 8, 150 mM NaCl, 1 g/L Proteinase K, 5% (v/v) 2-mercaptoethanol, 20 g/L SDS, 30 g/L PVP and 20 g/L activated charcoal; proteinase K and 2-mercaptoethanol were added just before use; SDS, PVP, and activated charcoal were not added to the buffer but rather ground with the fruit.) The powder was mixed into the extraction buffer, and the vial was then incubated at 65 °C for 20 min with mixing by inversion every 2 min during that time. After incubation, the vial was centrifuged (5 min at 20,000*g* and 25 °C), resulting in three phases (oil, aqueous, and solids). The reddish aqueous phase was extracted twice with 0.5 vol of phenol/chloroform/isoamyl alcohol (25:24:1). To the resulting still reddish aqueous phase, 1 vol of chloroform/isoamyl alcohol (24:1) and 10 mg of activated charcoal were added, mixed well, and separated by centrifugation (2 min @ 20,000*g* and 25 °C). The aqueous phase was transferred to a new vial and extracted with 1 vol of diethyl ether, resulting in the formation of a small amount of white precipitate, which was removed by centrifugation (2 min @ 20,000*g* and 25 °C). Precipitation of the nucleic acids in the sample was carried out by adding 5 M NaCl to a final concentration of NaCl of 200 mM and 1 vol of 2-propanol, followed by incubation at −20 °C for 20 min and subsequent centrifugation at 20,000*g* and 4 °C for 10 min. The resulting white pellet with black speckles (charcoal) was washed two times with 1 mL of 70% (v/v) ethanol and was dried at 30 mbar and 25 °C for 5 min. Resolvation of the pellet was done in 200 μL of TE (10 mM Tris–HCl, 1 mM Na_2_EDTA pH 8) by mixing with the pipet tip and incubating at 56 °C for 5 min. After redissolving the pellet, 20 μg of DNase-free RNase A was added, followed by another incubation for 5 min at 56 °C. The RNase-treated solution was extracted with 200 μL of phenol/chloroform/isoamyl alcohol (25:24:1) followed by a second extraction with 200 μL of chloroform/isoamyl alcohol (24:1). The now only slightly pinkish aqueous solution was centrifuged (2 min at 20,000*g* and 4 °C) which removed the last of the carried-over charcoal as a small black pellet. The supernatant was subjected to precipitation by ethanol (+100 mM NaCl, +2.5 vol ethanol), was stored at −20 °C for 30 min, and the precipitated nucleic acids were collected by centrifugation (10 min @ 20,000*g* and 4 °C). The off-white pellet was washed two times with 1 mL of 70% (v/v) ethanol each, dried at 30 mbar and 25 °C for 5 min, and finally dissolved in 100 μL of TE by mixing with a pipet tip and warming to 60 °C until all solids had disappeared.

DNA sequences of candidates for the gene encoding the isolated PPO were identified based on matches of the tryptic peptides generated from the purified protein to putative proteins of *O. europaea* cv. Farga.^11^ Primer pairs targeting these genes were designed (Table S1) and used to amplify the candidate genes from the olive DNA with Q5 High-Fidelity DNA Polymerase (New England Biolabs) following the manufacturer’s recommendations.

Amplification in the presence of 1× Q5 High GC Enhancer (New England Biolabs) yielded strong, clear bands for all tested annealing temperatures (58–70 °C). The amplicons were purified by agarose gel electrophoresis and subsequent isolation from the gel using a silica-column-based kit (Wizard SV Gel and PCR Clean-Up System from Promega). Purified amplicons were cut-ligated into the destination plasmid pEntryA (plasmid pENTRY-IBA51 from IBA Lifesciences with its kanamycin resistance marker replaced by an ampicillin resistance gene) using the type IIS restriction endonuclease SapI and T4 DNA ligase.^23^ The resulting plasmids were transformed into chemically competent *Escherichia coli* Top10 cells, selected on LB-agar plates with 100 mg/L ampicillin, isolated from a culture of the respective bacteria (Pure Yield Plasmid Miniprep System from Promega) and finally the sequence of the cloned genes (Table S2; Figure S2) was determined by Sanger sequencing (carried out by Microsynth GmbH, Vienna, Austria).

### Mass Determination

The purified protein was analyzed using a Dionex UltiMate 3000 Nano LC-system coupled to a Q Exactive mass spectrometer from Thermo Fisher Scientific, equipped with a Nanospray ion source. Before conducting mass spectrometry measurements, the purified *Oe*PPO solution underwent ultrafiltration (30 kDa cutoff) through centrifugation, and the buffer solution was changed to 5 mM ammonium acetate at pH 7.0. Samples were first loaded onto a μ-precolumn (trap column) PepMap300 (Thermo Scientific, 300 μm i.d. × 5 mm, C4, 5 μm, 300 Å) using a solvent mixture of 2% (v/v) acetonitrile, 97.9% (v/v) H_2_O, and 0.1% (v/v) trifluoroacetic acid, operating at a flow rate of 10 μL/min. Subsequent separation took place on an Accucore C4 analytical column (Thermo Scientific, 75 μm × 50 cm, nanoViper C4, 2.6 μm, 150 Å) with a flow rate of 300 nL/min. Full-scan mass spectra were recorded in positive ion mode, ranging from 600 to 2500 *m*/*z*, with a resolution of 17.5k for proteins larger than 20 kDa and from 500 to 2500 *m*/*z* with a resolution of 70k for proteins smaller than 20 kDa. The electrospray voltage applied was 2.2 kV, and the ion transfer capillary temperature was maintained at 300 °C. Postacquisition, the acquired data were processed using Thermo Xcalibur 3.0.63 Qual Browser.

### Data Analysis

All experiments were conducted in triplicate, and the results are presented as means with corresponding standard deviations. Data processing and visualization were performed by using XLSTAT 2009 and OriginPro 2023b, respectively.

## Results and Discussion

### Extraction and Purification of *Oe*PPO

The extraction of purifiable proteins from olive fruits posed significant challenges. While standard protocols that have proven to be effective for many plant PPOs were tested during the preliminary experiments, they did not yield successful results for olives. The presence of several interfering compounds, such as oil, pectins, and phenolics, complicated the extraction process.^13^ As a result, we adopted the protocol described above that effectively addressed the aforementioned issues. This novel approach involved a combination of acetone precipitation and repeated aqueous two-phase separation (ATPS). By employing acetone precipitation, a significant portion of the phenolics and oil was successfully washed out. Subsequently, the residual interfering compounds were quantitatively removed from the crude extract through a series of steps, which involved repeated Triton X-114-induced ATPS, ammonium sulfate precipitation, and iterated PEG-4000-induced ATPS. Additionally, the gradual addition of ammonium sulfate to 30% saturation further facilitated the precipitation of interfering compounds, thereby reducing their presence. The remaining interfering compounds were effectively eliminated via PEG-4000-induced ATPS, carefully avoiding protein precipitation by using descending additions of PEG-4000.^14^ This meticulous process culminated in the acquisition of a clear and enzymatically active solution.

The crude extract underwent two rounds of dialysis mainly to remove salts, small nontarget proteins, phenolics, and ascorbate (second round). Ascorbate was included in the first round to avoid browning due to the possible presence of traces of phenolics in the crude extract. Subsequently, the resulting protein solution was purified using ion exchange chromatography with an FPLC system, where the enzyme solution was applied first to an anion-exchange column. During this chromatography, two major protein peaks were eluted. The highest *Oe*PPO activity was found in the fractions recovered from the first peak, eluted at ∼20 mS/cm (Figure 1A). Insignificant activity was observed in the majority of the fractions eluted from the second peak. The SDS-PAGE gel showed that this first column had already purified one major protein along with a few other minor proteins (Figure 1C). The active fractions (369–422 mL) were pooled, concentrated, and then purified by cation exchange chromatography on a Mono S column, which resulted in the elution of the protein into a single peak of *Oe*PPO activity at ∼1 mS/cm (4–7 mL) (Figure 1B). With this final chromatographic step, a successful purification of *Oe*PPO was achieved, with a 17-fold, 63% recovery rate and a specific activity of 204 U/mg using 10 mM catechol (Table 1). *Oe*PPO appeared as a single band on an SDS-PAGE gel at ∼35 kDa (Figure 1C). Despite attempts to separate the purified *Oe*PPO from possible isoforms, using additional chromatographic steps, the purified *Oe*PPO consistently eluted as a single peak (data not shown).

**Table 1:** Purification of Polyphenol Oxidase from Olive

purification stage	volume (mL)	total protein (mg)	total activity^a^ (units)	specific activity (units/mg protein)	purification (fold)	yield (%)
crude extract^b^	150	147.0 ± 2.3	1741 ± 67	11.9 ± 0.5	1.00	100
dialysis	154	64.7 ± 1.9	1658 ± 35	25.6 ± 0.5	2.15	95.2
anion exchange	53	15.9 ± 0.7	1348 ± 20	84.8 ± 1.3	7.13	77.4
cation exchange	3	5.4 ± 0.1	1101 ± 8	203.9 ± 1.6	17.13	63.2

a Enzymatic activity was determined on 10 mM catechol in 50 mM phosphate buffer, pH 7.5.

b The crude extract was obtained from 250 g of pitted olives.

### Gel Electrophoresis and Molecular Weight Determination

**Figure 2:**
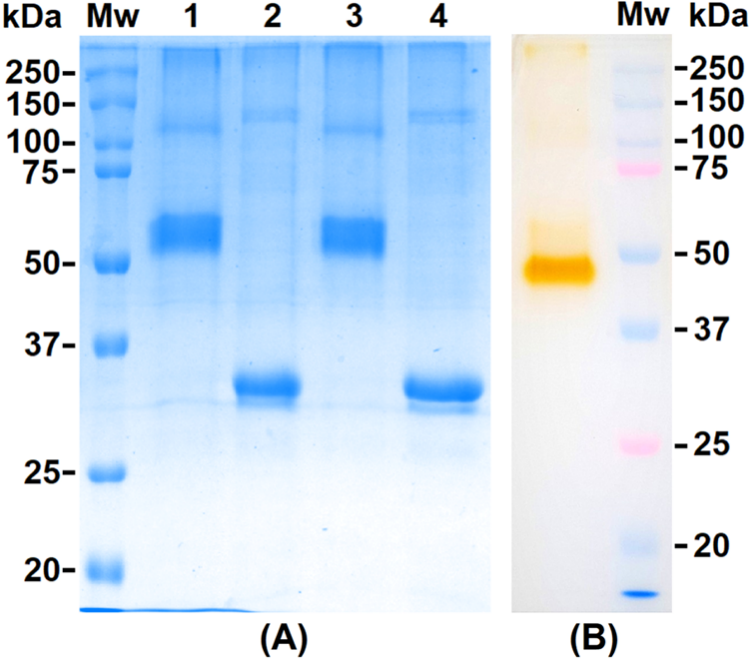
SDS-PAGE of *Oe*PPO (15 μg). (A) SDS-PAGE stained with Coomassie blue of *Oe*PPO: (1) nonheated + reduced, (2) heated + reduced, (3) nonheated + nonreduced, and (4) heated + nonreduced. (B) Activity gels of purified *Oe*PPO (nonreduced + nonheated) stained with catechol.

The purified *Oe*PPO, obtained from cation exchange chromatography, was analyzed by denaturing SDS-PAGE stained with Coomassie brilliant blue G-250. The enzyme appeared as a single band at approximately 35 kDa (Figure 1C). This molecular weight falls within the reported range (32–200 kDa) for plant PPOs.^4^ However, it is significantly lower than the molecular weight (∼55 kDa) reported by Sánchez et al.^11^ for the mature *Oe*PPO. Additionally, the confirmed latency of the purified enzyme using SDS excludes the possibility that the enzyme was purified in its active form. Therefore, in-gel enzymatic activity staining was performed with catechol as the substrate to visualize enzyme activity. Surprisingly, the results (Figure 2B) revealed the appearance of a strong activity band at around 49 kDa. Notably, no band was observed at a molecular weight of 35 kDa, as previously seen on the denaturing SDS-PAGE gel.

To investigate the discrepancy between the apparent molecular weight of the purified *Oe*PPO on denaturing SDS-PAGE and the molecular weight reported on activity gel (in-gel enzymatic activity staining), we performed another SDS-PAGE experiment using four distinct conditions: (1) nonheated + reduced; (2) heated + reduced; (3) nonheated + nonreduced; and (4) heated + nonreduced. Coomassie blue staining was used for visualization. The results (Figure 2A) revealed that the choice of denaturing SDS-PAGE conditions influenced the appearance of the enzyme on the gel. In the nonheated samples (Figure 2A, lines 1 and 3), the enzyme appeared as a single thick band at around 55 kDa. This mass matches the reported mass (∼55 kDa) for the mature protein as documented by Sánchez et al.^11^ The electrophoretic profile of *Oe*PPO on the SDS-PAGE gel, appearing as a thick band (double band), has also been observed in pure recombinant *Oe*PPO1.^11^ This characteristic electrophoretic pattern has been reported for other plant PPOs as well.^24^ The presence of *Oe*PPO as a double band suggests potential modifications in the enzyme’s folding^11,24^ or the possible existence of multiple isoforms of the enzyme that share similar physicochemical properties but differ slightly in their molecular weight. In contrast, the heated samples appeared as a single protein band at ∼35 kDa (Figure 2A, lines 2 and 4), suggesting that the heat treatment step, rather than the reducing agent, is responsible for the discrepancy between the two apparent molecular weights. These observations suggest that the denaturation triggered by heat not only impacts the enzyme’s tertiary structure but also prompts the latent *Oe*PPO to unfold into a smaller protein. This suggests that the mature protein is oligomeric or the C-terminal domain was cleaved from the main core of a monomeric enzyme, resulting in the formation of a denatured tyrosinase domain with an approximate molecular weight of ∼35 kDa. In the context of an oligomeric protein, the attachment of the C-terminal domain/subunit of *Oe*PPO to the main domain might involve nonpeptide bonds such as hydrophobic bonds and polar interactions.^25^ Nonpeptide binding of the C-terminal domain to the main domain was also observed in the PPO from *Coreopsis grandiflora* petals.^14^ In this instance, the linkage between the C-terminal domain and the main domain was accomplished through the presence of disulfide bridges.^14^ However, in our case, the possibility of disulfide bridges between the two domains is excluded, as reducing conditions did not impact the enzyme’s appearance on SDS-PAGE gel. Our findings are consistent with the observations made by Ortega-García et al.^12^ In their study, the authors employed a nonpurified olive protein extract to stain an activity gel for olive PPO during olive fruit maturation. They noted the emergence of two distinct activity bands: one at 55 kDa and another at 36 kDa. The latest band at 36 kDa was observed to emerge during the later stages of maturation.^12^ This indicates that the band emerging in our case at 35 kDa is mostly active *Oe*PPO after C-terminal cleavage/dissociation.

On the other hand, the appearance of *Oe*PPO at two distinct positions on the activity gel (49 kDa) and the Coomassie blue-stained gel (54 kDa) is common for PPOs. Active PPO, when subjected to activity staining on a gel, appears at a lower molecular weight band compared to its position on Coomassie blue-stained gel.^26−30^ The purified enzyme displays distinct electrophoretic mobility under the two conditions, with active PPO potentially having increased mobility due to intact intramolecular bridges stabilizing its conformation.^26,31^ Moreover, unlike denatured and fixed proteins in Coomassie-stained gels, unfixed active proteins in activity-staining gels are not entirely restricted to their initial positions and may undergo additional migration, resulting in a shift from their original locations.^31^

### Copper Ion Content

The quantification of copper ion content in *Oe*PPO was accomplished spectrophotometrically by measuring the absorption at 546 nm (with ε = 6300 M^–1^ cm^–1^) of a Cu(I)-2′-biquinoline complex.^17^ The results revealed that each *Oe*PPO molecule contains an approximate count of 1.88 ± 0.07 copper ions. This value strongly suggests the presence of both copper ions (CuA and CuB) in the active centers of nearly all of the enzyme molecules. The minor variance from the theoretical value could potentially be attributed to trace contamination of the purified enzyme solution by negligible amounts of non-PPO proteins. Alternatively, this discrepancy could be attributed to the possible loss of copper ions during the extraction and purification processes or to PPOs that were initially not fully loaded.

### Effect of SDS

**Figure 3:**
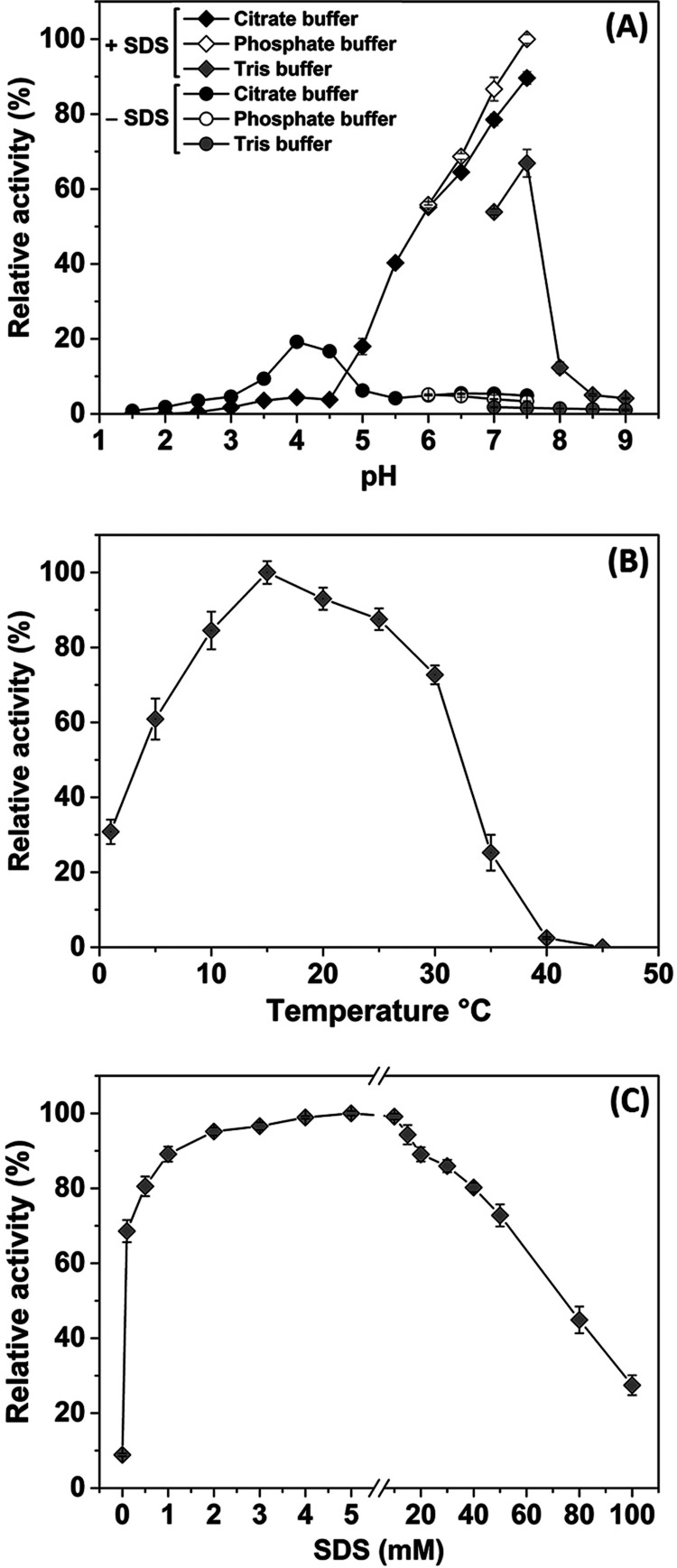
Effects of pH, temperature, and SDS on *Oe*PPO activity. (A) The effect of pH on *Oe*PPO activity: in the absence (−SDS) and presence (+SDS) of 5 mM SDS. (B) Effect of temperature on *Oe*PPO activity. (C) The effect of SDS on *Oe*PPO activity.

The effect of ascending SDS concentrations on *Oe*PPO activity was investigated under normal assaying conditions in a phosphate buffer with a pH of 7.5 (Figure 3C). *Oe*PPO displayed weak but detectable activity in the absence of SDS. However, the addition of 0.1 mM SDS significantly increased *Oe*PPO activity. The enzymatic activity showed a proportional increase with the concentration of SDS, peaking at 5 mM (∼10 fold). Beyond this concentration, the *Oe*PPO activity started to decrease slowly. Nevertheless, *Oe*PPO maintained considerable activity, even with 100 mM SDS, exhibiting three times the activity compared to the absence of SDS. These results strongly indicate that *Oe*PPO was purified in its latent form. The optimal concentration required for full activation of latent PPO (5 mM) is remarkably higher than the concentrations reported for other plant PPOs,^4,10^ such as banana^32^ and apricot^33^ (2 mM). The exception is mango^34^ PPO, which exhibits an even higher requirement, with 6.93 mM necessary to achieve full activity. The decrease in PPO activity beyond the optimal concentration of SDS has been observed in other plant PPOs as well.^32,33^ However, olive PPO appears to exhibit resistance to relatively high SDS concentrations compared to apricot^33^ and banana PPOs.^32^

### Effect of pH

The influence of pH on *Oe*PPO activity was measured at different pH values, ranging from 1.5 to 9.0, using catechol in the presence and absence of 5 mM SDS as an activator (Figure 3A). In the presence of SDS, *Oe*PPO activity was very weak (<5%) at low pH levels (≤4.5), and practically no activity was observed at pH 1.5 and 2.0. However, as the pH increased above 5.0, the *Oe*PPO activity started to rise, reaching its optimal activity at pH 7.5. Beyond this pH (7.5), *Oe*PPO activity dropped dramatically in the alkaline region, with only 12.3 and 5.0% relative activity reported at pH 8.0 and 8.5, respectively. At its optimum pH, *Oe*PPO showed the highest relative activity with phosphate buffer (100%), followed by citrate buffer (89.6%) and Tris buffer (66.9%). Enzymatic activity can vary significantly when tested in different buffer systems, even if they share the same pH and concentration.^35^ Components of the buffer, through their ionic strength and nature, can directly influence the catalytic process of the enzyme and its activity.^35^ The optimal pH value (7.5) in the presence of SDS is slightly higher than the reported optimal pH of 7.0 for recombinant *Oe*PPO1.^11^

On the other hand, in the absence of SDS (Figure 3A), *Oe*PPO displayed a preference for acidic pH, remaining active even at pH 1.5 with 0.8% relative activity. The enzyme’s activity peaked at pH 4.0, showing a relative activity of 19.2%, which was five times higher than its activity in the presence of SDS (4.4%). The optimal pH values for our enzyme, both in the presence and absence of SDS, fall within the range of 4.0–8.0, as reported by Yoruk and Marshall^4^ for plant PPOs. Latent PPO is well reported to exhibit activation in response to acidic pH.^4,10,33,36,37^ Therefore, the pH optimum reported in the absence of SDS is attributed to the acidic activation of the latent enzyme. Similar observations were reported for apple,^37^ apricot,^33,38^ peach, plum, and cherry PPOs,^38^ with an acidic optimal pH of 4.0–5.0 in the absence of SDS and an optimum pH of 6.0–7.0 when activated with SDS.

The occurrence of two distinct optimal pH values for latent PPO in the presence and absence of SDS is a common observation for plant PPOs, as also reported elsewhere.^10,33,37,38^ Notably, the activity at the optimal pH (usually neutral) in the presence of SDS is significantly higher than the activity at the acidic pH optimum in the absence of SDS. The addition of SDS causes a shift in the pH optimum of the enzyme from low to higher pH values.^4,10,38^ The two activation conditions differ considerably in their effectiveness, with the presence of the activator (SDS) and optimal ionization state of the enzyme enhancing PPO activity more than acidic activation alone.^38^

### Effect of Temperature

The effects of temperatures ranging from 1 to 45 °C on *Oe*PPO activity are presented in Figure 3B. The results demonstrate that *Oe*PPO exhibits activity even at 1 °C, with 30% relative activity. As the temperature increases, the activity rises, reaching its maximum at 15 °C, after which it gradually declines with further temperature increase. Surprisingly, the enzyme becomes completely inactive at 45 °C. These findings indicate that *Oe*PPO is a cold-adapted enzyme (psychrophilic), and its catalysis of substrates is highly thermosensitive compared to other plant PPOs. This temperature remains below the optimal temperatures typically reported for plant PPOs,^4,8^ which usually fall within the range of 25–45 °C.^4^ Notably, the Jackfruit PPO^39^ stands as an exception, exhibiting an even lower temperature of 8 °C, using catechol and 4-methylcatechol as substrates. Diverging from the maturation pattern of many Mediterranean fruits, olives undergo their maturation process from late fall to early winter in a relatively colder environment (∼10 °C). It is well established that enzymes across various organisms have adapted to perform optimally within the temperature conditions of their native surroundings.^40,41^ This optimal temperature range tends to align closely with the customary temperature range of the fruit’s natural habitat. This correlation could potentially elucidate the rationale behind the lower temperature range at which olive polyphenol oxidase (*Oe*PPO) exhibits its peak activity.

In our study, we preferred the use of 25 °C for measuring *Oe*PPO activity. Most protocols recommend utilizing 25 °C (room temperature) as the assay temperature^35^ since it mitigates temperature fluctuations, trims experimental time, and diminishes both sample volume and enzyme usage, particularly when employing a microplate reader, in contrast to assessing enzymatic activity at 15 °C (optimal temperature), which mandates switching from the microplate reader to the Shimadzu spectrophotometer linked to a thermostat via a circulating water bath.

### Thermal Stability

**Figure 4:**
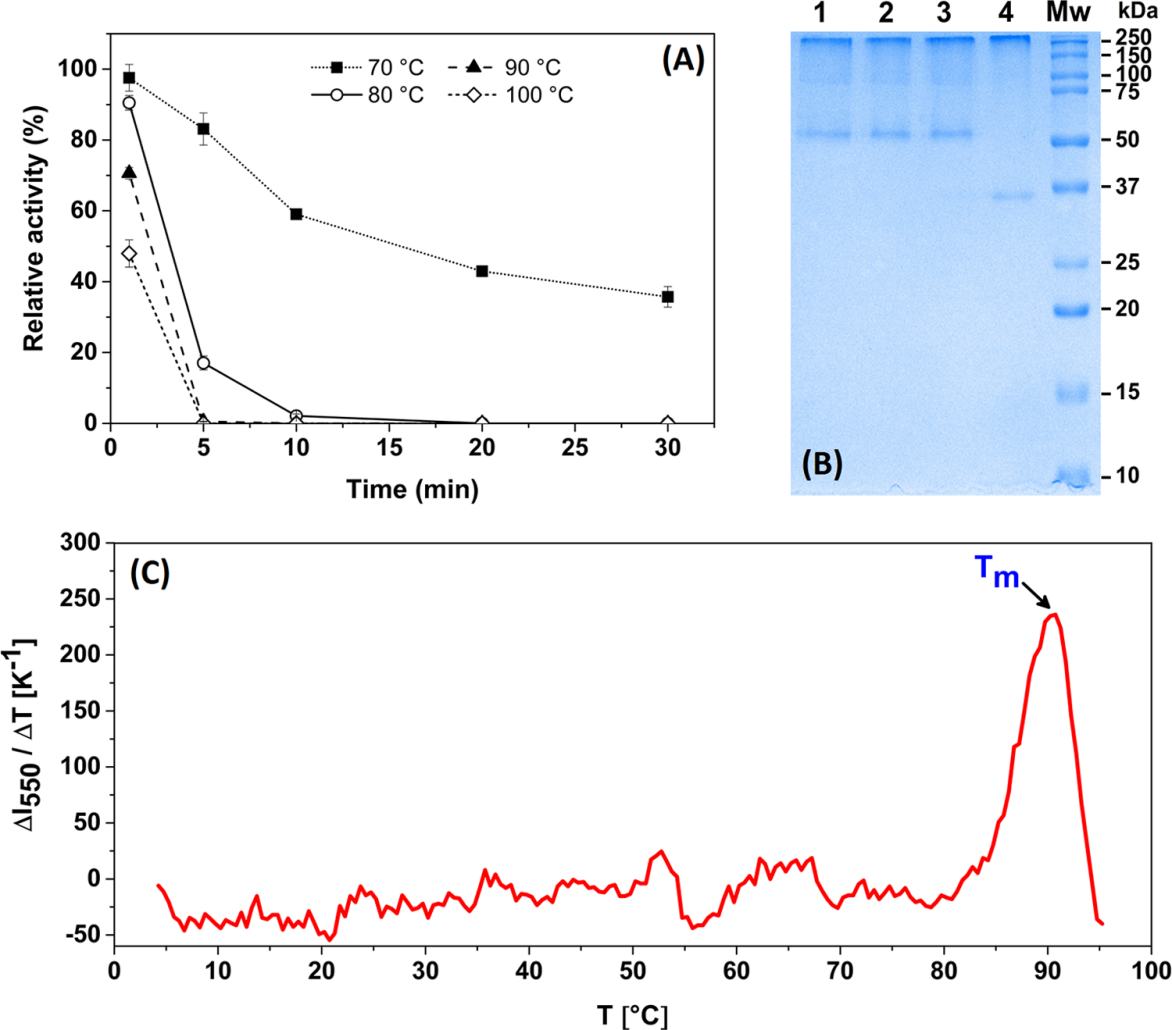
Thermal stability of *Oe*PPO. (A) The effect of preincubation temperature on *Oe*PPO activity. The enzyme activity was measured using 0.5 μg of *Oe*PPO and 10 mM of catechol as a substrate. (B) The effect of preincubation temperature on *Oe*PPO (5 μg) appearance on SDS-PAGE. (1) Preincubation at 70 °C/5 min, (2) preincubation at 80 °C/5 min, (3) preincubation at 90 °C/5 min, (4) preincubation at 100 °C/5 min, and (Mw) molecular weight marker (values are given in kDa). (C) Thermal shift assay of *Oe*PPO. The melting temperature value (*T*_m_) was determined from the derivative of fluorescence intensity (*I*, in arbitrary units) with respect to the temperature increment. The raw fluorescence intensities are shown in Figure S3.

The thermal stability profile for *Oe*PPO, assessed based on the residual percentage activity, is depicted in Figure 4A. *Oe*PPO exhibited relative heat resistance, showing residual activity of 2.10 and 35.7% at 80 °C/10 min and 70 °C/30 min, respectively. *Oe*PPO was only completely inactivated after 5 min of heat treatment at 90 and 100 °C, or 20 min at 80 °C. These results were intriguing because the optimal activity of the enzyme was found at 15 °C, and it was entirely inactive at 45 °C, indicating that the enzyme is heat resistant but requires low temperatures for activity.

Typically, elevated temperatures enhance PPO activity until a critical point, causing concurrent activation and inactivation processes. Inactivation predominating at higher temperatures results in a drop in residual activity linked to enzyme tertiary structure unfolding.^35^ However, this pattern was not fully observed in the case of *Oe*PPO. Hence, to gain a deeper insight into the enzyme’s thermal stability, a thermal shift assay was conducted (Figures 4C and S3). The assay revealed a single melting temperature (*T*_m_) of 90 °C for purified *Oe*PPO. The unfolding of *Oe*PPO at 90 °C corroborated the results of the thermal stability assessment and suggested that denaturation occurred primarily at ∼90 °C. Notably, the resulting *T*_m_ for *Oe*PPO was higher than the *T*_m_ reported for dragon-eye fruit (*Dimocarpus longan*) PPO,^42^ which exhibited three lower melting temperatures at 54.5, 70.5, and 81.5 °C.

The thermal shift assay results, along with previous observations of denaturing and nondenaturing SDS-PAGE gels, raised our suspicion that the unfolding event at 90 °C was the primary cause of OePPO transitioning from the oligomeric to the monomeric form. To substantiate this, we conducted a second SDS-PAGE analysis using four denaturing temperatures (70, 80, 90, and 100 °C) for 5 min. After subjecting the enzyme to heat treatment, a single band was observed at 70 and 80 °C, while at 90 °C, a strong band at 55 kDa and a weaker band at 35 kDa were displayed. At 100 °C, the enzyme appeared as a single band at 35 kDa. These findings provided robust evidence to support our initial hypotheses, confirming that the denaturation occurring at 90 °C is due to the irreversible unfolding of the enzyme into its monomeric form of 35 kDa. Given that the enzyme’s natural structure is oligomeric, temperatures above 45 °C seem to cause only a reversible disruption of the structure, significantly reducing its substrate binding and catalytic ability. Thus, once the temperature is lowered, the enzyme regains most of its activity. At 90 °C, however, the oligomeric structure breaks down into an inactive monomeric form, resulting in complete denaturation of the enzyme. These observations indicate that as long as *Oe*PPO preserves its oligomeric structure, it remains remarkably stable. Therefore, the notable stability of *Oe*PPO is likely a result of its oligomeric structure. Several research papers showed that enzymes and proteins consisting of multiple subunits generally display enhanced thermal stability.^43−45^ The interactions between different subunits are believed to play a pivotal role in significantly enhancing the overall stability of proteins. Furthermore, the dissociation of subunits within oligomeric proteins is often recognized as the primary event in the deactivation pathway.^46^

### Enzyme Kinetics and Substrate Specificity

Enzyme kinetics and substrate specificity (Table 2, Figure S4) of *Oe*PPO were thoroughly examined using two monophenols (tyramine and tyrosol) and six diphenols: four of which are classic PPO substrates (catechol, 4-methylcatechol, dopamine, 4-*tert*-butylcatechol), and two are the most abundant natural phenolics found in olive fruits (hydroxytyrosol and oleuropein). *Oe*PPO activity was assayed under the same conditions reported previously, except for varying substrate concentrations. For a more accurate estimation of kinetic parameters (*K*_m_ and *k*_cat_), nonlinear regression was preferred over Lineweaver–Burk linearization, as the latter is more sensitive to experimental errors and tends to overemphasize the impact of low substrate concentrations. The *k*_cat_/*K*_m_ ratio was then utilized to evaluate the enzyme’s substrate specificity.

**Table 2:** Kinetic Parameters of Purified *Oe*PPO

substrate^a^	λ_max_ (nm)	ε_max_ (M^–1^ cm^–1^)	*K*_m_ (mM)	*k*_cat_ (s^–1^)	*k*_cat_/*K*_m_ (s^–1^ mM^–1^)
Monophenols					
tyramine	480	3300	0.88 ± 0.14	0.093 ± 0.005	0.107 ± 0.012
tyrosol	399	1225	2.23 ± 0.04	0.997 ± 0.005	0.448 ± 0.008
Diphenols					
oleuropein	402	1190	1.66 ± 0.07	18.11 ± 0.72	10.93 ± 0.27
hydroxytyrosol	402	1205	1.34 ± 0.06	47.41 ± 0.11	35.45 ± 1.54
catechol	410	1623	6.53 ± 0.27	119.97 ± 1.78	18.36 ± 0.53
4-methylcatechol	400	1638	3.22 ± 0.11	77.09 ± 0.56	23.91 ± 0.66
4-*tert*-butylcatechol	400	1200	3.13 ± 0.05	55.56 ± 0.61	17.73 ± 0.11
dopamine	480	3300	12.10 ± 0.88	13.98 ± 0.07	1.16 ± 0.08

a The chemical structures of the tested substrates are available in the SI (Figure S1).

The purified *Oe*PPO sufficiently catalyzed the hydroxylation and oxidation of two monophenolic substrates (tyramine and tyrosol); therefore, it was identified as a tyrosinase (EC 1.14.18.1) with the ability to hydroxylate monophenols and oxidize diphenols. Interestingly, tyrosol was processed faster than tyramine (>10 times faster), although tyramine showed the lowest *K*_m_ value among the tested substrates (0.88 ± 0.14 mM). However, the monophenolase activity was relatively low compared to the diphenolase activity. PPOs from many plants, such as apricots,^36^ apples,^37^ and dragon-eye fruit,^42^ were also identified as tyrosinases. Nonetheless, the monophenolase activity in plant PPOs is generally weaker when compared to fungal tyrosinases.^5^ The kinetics of *Oe*PPO toward diphenolic compounds revealed that catechol, 4-methylcatechol, and 4-*tert*-butylcatechol are the most rapidly processed phenolics, with *k*_cat_ values of 119.97 ± 1.78, 77.09 ± 0.56, and 55.56 ± 0.61 s^–1^, respectively, followed by the substrates occurring naturally in olives: hydroxytyrosol (*k*_cat_ = 47.41 ± 0.11 s^–1^) and oleuropein (*k*_cat_ = 18.11 ± 0.72 s^–1^). On the other hand, dopamine exhibited the lowest *k*_cat_ value (13.98 ± 0.07 s^–1^) and the highest *K*_m_ value (12.10 ± 0.88 mM), resulting in a very low catalytic efficiency (*k*_cat_/*K*_m_ = 1.16 ± 0.08 s^–1^ mM^–1^) compared to other substrates. Conversely, the highest catalytic efficiency (*k*_cat_/*K*_m_ = 35.45 ± 1.54 s^–1^ mM^–1^) among the tested substrates was observed for the substrate hydroxytyrosol (occurs naturally in olives), which also has the lowest *K*_m_ value (1.34 ± 0.06 mM) among the diphenols. These findings suggest that hydroxytyrosol is likely one of the main physiological substrates responsible for the browning of olive fruits. Additionally, the results imply that oleuropein could also be one of the preferred phenolic compounds targeted by *Oe*PPO *in vivo*. The high catalytic efficiency of PPO toward indigenous substrates, as compared to classic substrates, was also observed in the cases of apricot^33^ and dragon-eye fruit^42^ PPOs.

### Effect of Inhibitors

**Figure 5:**
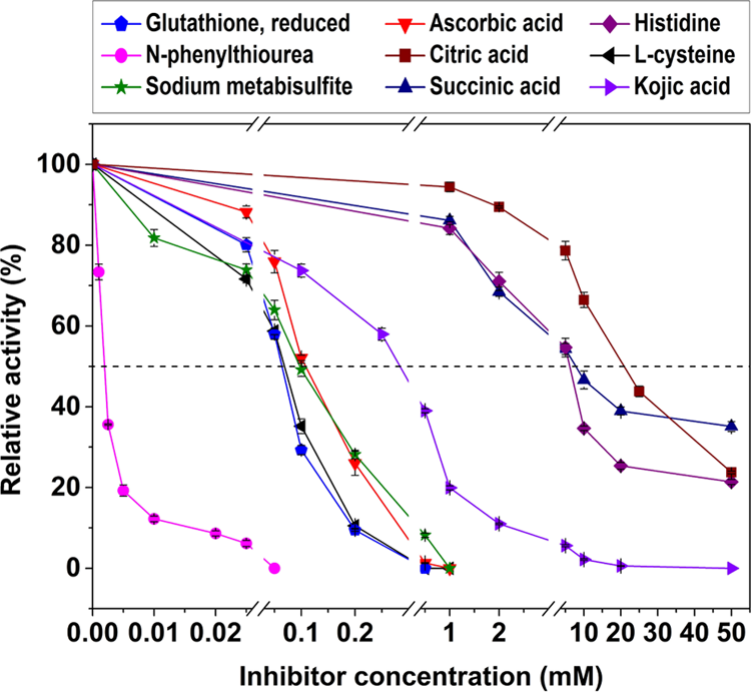
Effects of inhibitors on *Oe*PPO activity.

The impact of various inhibitors on the *Oe*PPO activity was investigated (Figure 5). The findings indicated that *N*-phenylthiourea is the most effective inhibitor, as it completely inhibited enzymatic activity at a concentration of 50 μM, while a mere 1.8 μM was sufficient to inhibit 50% of the *Oe*PPO activity (Table 3). Alongside this notable inhibitor, ascorbic acid, reduced glutathione, sodium metabisulfite, and l-cysteine exhibited substantial inhibitory capabilities, displaying comparable inhibitory profiles with IC_50_ values ranging from 0.062 to 0.11 mM (Table 3). At a higher concentration of 1 mM, these inhibitors demonstrated complete suppression of the *Oe*PPO activity. Kojic acid demonstrated moderate inhibitory potential relative to the other inhibitors, exhibiting an IC_50_ value of 0.31 mM. However, to achieve complete inhibition, a higher concentration of 50 mM was required. Histidine, citric acid, and succinic acid, on the other hand, were identified as the weakest inhibitors. Despite exposure to 50 mM concentrations of these inhibitors, residual enzymatic activity of 21.36 ± 0.62, 23.71 ± 0.33, and 35.08 ± 1.23, respectively, persisted, underscoring their limited effectiveness in hindering *Oe*PPO activity.

**Table 3:** Half-Maximal Inhibitory Concentration of Tested Inhibitors

inhibitors	IC_50_ (mM)^a^
ascorbic acid	0.11 ± 0.008
citric acid	18.30 ± 1.02
glutathione, reduced	0.062 ± 0.003
histidine	5.97 ± 0.41
Kojic acid	0.306 ± 0.022
l-cysteine	0.0656 ± 0.0047
*N*-phenylthiourea	0.0018 ± 0.0002
sodium metabisulfite	0.081 ± 0.005
succinic acid	9.78 ± 0.66

a IC_50_, half-maximal inhibitory concentration (determined with nonlinear regression).

Ascorbic acid, reduced glutathione, sodium metabisulfite, and l-cysteine have been identified as effective inhibitors for several plant PPOs, exhibiting complete inhibitory effects at concentrations around 1 mM on PPOs from apricot,^36^ blueberry,^47^ and mango.^48^ Furthermore, aligning with our findings, kojic acid has exhibited a similar efficacy on mango PPO,^48^ effectively inhibiting enzymatic browning at a concentration of 10 mM. Furthermore, succinic acid and citric acid have been documented as less potent inhibitors. They exhibit weak inhibitory effects, resulting in residual activities exceeding 80% in apricot^36^ and surpassing 60% in mango,^48^ even at concentrations of 10 mM.

The inhibition of enzymatic browning encompasses a range of mechanisms that can act in isolation or synergistically.^4^ Among the inhibitors investigated, *N*-phenylthiourea predominantly exerts its inhibitory effect by binding to the binuclear copper at the active site of PPO. However, it also exhibits additional inhibitory actions, involving binding to other regions of the enzyme and interacting with quinones, leading to the suppression of melanin formation.^49^ Metabisulfite, ascorbic acid, l-cysteine, and kojic acid function as reducing agents and thwart enzymatic browning reactions by reacting with quinones, leading to the formation of a stable, colorless product.^4,50^ Moreover, metabisulfite’s inhibitory action extends to direct binding with the “*met*” and “*oxy*” forms of binuclear copper at the enzyme’s active site, thus modulating its function.^50^l-cysteine, on the other hand, can readily form complexes with quinones, thereby inhibiting secondary oxidation and polymerization reactions.^51^ Citric and succinic acids inhibit enzymatic browning through two primary mechanisms. First, acting as weak acids, they effectively lower the pH of the surrounding environment. Second, both citric acid and succinic acid demonstrate chelating properties, forming stable complexes with metal ions, particularly copper ions, that reside in the active site of the PPO enzyme.^4^ In a similar manner, histidine utilizes its imidazole group to bind to the copper ions present in PPO’s active site through coordination.^52^ This interaction leads to a disruption of the enzymatic activity of PPO, thereby impeding its ability to catalyze the oxidation of polyphenols.^53^ Despite these inhibitory mechanisms, it seems that *Oe*PPO exhibits a lower susceptibility to the inhibitory effects of citric acid, succinic acid, and histidine when compared to the other tested inhibitors.

Among the tested inhibitors, in order of efficacy, reduced glutathione, sodium metabisulfite, l-cysteine, and ascorbic acid can be considered to be effective in controlling enzymatic browning. The inhibition could be more effective if applied in conjunction with unfavorable conditions for *Oe*PPO activity, such as mild temperatures (35–45 °C) and a pH between 5.0 and 6.0 (as lower pH can activate the latent form of *Oe*PPO). While *N*-phenylthiourea might be an even more effective choice for enzymatic browning inhibition, it is not a common food additive; therefore, its safety as a food additive requires further studies. On the other hand, ascorbic acid stands out as the safest inhibitor for human consumption.^54^

### Effect of Metal Ions on Enzymatic Activity

The impact of various metal ions on the *Oe*PPO activity was investigated (Table 4). At 1 mM, most metal ions had a negligible effect, except for Cr^3+^ and Fe^2+^, which significantly reduced the residual activity to 80 and 87%, respectively. Positive changes were observed at 5 mM with Zn^2+^ and Cu^2+^, increasing the residual activity to 109 and 112%, respectively. At 10 mM, most metal ions decreased the *Oe*PPO activity, with Cr^3+^ causing complete suppression. At 100 mM, all tested metal ions drastically reduced the *Oe*PPO activity, including Zn^2+^ and Cu^2+^.

**Table 4:** Effect of Metal Ions on *Oe*PPO1 Activity

	relative activity (%)			
metal salt	1 mM	5 mM	10 mM	100 mM
AlCl_3_	98 ± 1.9	90 ± 1.2	82 ± 2.7	0.0 ± 0.00
CaCl_2_	103 ± 2.0	96 ± 2.8	93 ± 2.9	66 ± 4.1
CoCl_2_	102 ± 4.5	102 ± 2.5	95 ± 3.3	31 ± 1.2
CrCl_3_	80 ± 2.8	63 ± 1.6	0.0 ± 0.00	0.0 ± 0.00
FeCl_2_	87 ± 2.3	74 ± 1.7	56 ± 2.0	22 ± 6.6
KCl	98 ± 3.8	98 ± 1.4	96 ± 2.1	64 ± 2.8
MgCl_2_	100 ± 2.7	97 ± 1.4	95 ± 1.8	72 ± 3.4
MnCl_2_	99 ± 2.1	92.1 ± 0.84	60 ± 3.2	0.0 ± 0.00
NaCl	101 ± 3.1	99 ± 2.3	103 ± 2.8	88 ± 5.4
ZnCl_2_	99 ± 1.2	108.9 ± 0.92	107.3 ± 0.54	15 ± 5.1
CuSO_4_	106 ± 4.3	112 ± 1.9	110 ± 3.9	37 ± 2.6
NiSO_4_	103 ± 2.3	101.3 ± 0.57	92 ± 1.7	88 ± 4.3

Metal ions exhibit the capability of modulating enzymatic activity through various mechanistic pathways. This modulation can be both positive and negative, and it often depends on the type and concentration of the metal ions involved. Notably, metal ions can engage in competitive interactions with substrates for binding to the enzyme’s active site.^55,56^ Additionally, metal ions can bind to the allosteric sites of the enzyme, thereby prompting conformational alterations that induce allosteric inhibition.^55,56^ Furthermore, certain metal ions can induce a deficiency of copper ions within polyphenol oxidases (PPOs) by replacing copper ions within the enzyme’s active center.^56^ Distinct metal ions, on the other hand, may promote the PPO activity. Among these, copper ions can increase the rate of phenolic compound oxidation by facilitating electron transfer during the reaction.^56^ The induction of PPO activity in the presence of copper ions has been documented across numerous plant PPOs, encompassing apple^57^ and apricot.^36^ Similarly, zinc ions have been reported to enhance PPO activity, including instances such as apricot^36^ and mango.^48^ Since copper ions are indispensable for PPO function, the artificial addition of copper ions to the environment in which the enzyme exists will exert a consequential influence on PPO activity. By binding to the active site of the enzyme, they actively partake in the oxidation of phenolic compounds, thereby orchestrating a discernible increase in enzymatic activity. However, high concentrations of metal ions can lead to denaturation of the enzyme, effectively perturbing its structural integrity and causing a loss of activity.^55,56^ Furthermore, elevated salt concentrations have the potential to amplify hydrophobic interactions, resulting in the aggregation of enzymes and subsequent reduction in their activity.^58^ Additionally, an excessive presence of metal ions can lead to the repulsion of oxygen, consequently inhibiting PPO activity.^58^

### Protein Identification, Sequence Confirmation, and Molecular Mass Determination

PPO can exist in multiple variants in olive fruits, with the sequence of these variants showing significant differences between various cultivars.^11^ Furthermore, these variants display notable distinctions in biochemical properties to the extent that the enzyme may either lack or possess monophenolase activity.^11^ Therefore, for the accurate identification of our purified enzyme and to facilitate the interpretation of mass spectrometry results, it was necessary to clone *Oe*PPO genes and obtain the gene that encodes the isolated protein, as the sequences present in the database are from different olive cultivars. The cloned genes of *Oe*PPO from cv. Chemlal (Table S2) have unveiled the existence of six potential *Oe*PPO variants in olive fruit DNA. The cloned sequences are presented in Table S2 and comparatively aligned with the variants identified by Sánchez et al.^11^ in Figure S2.

**Figure 6:**
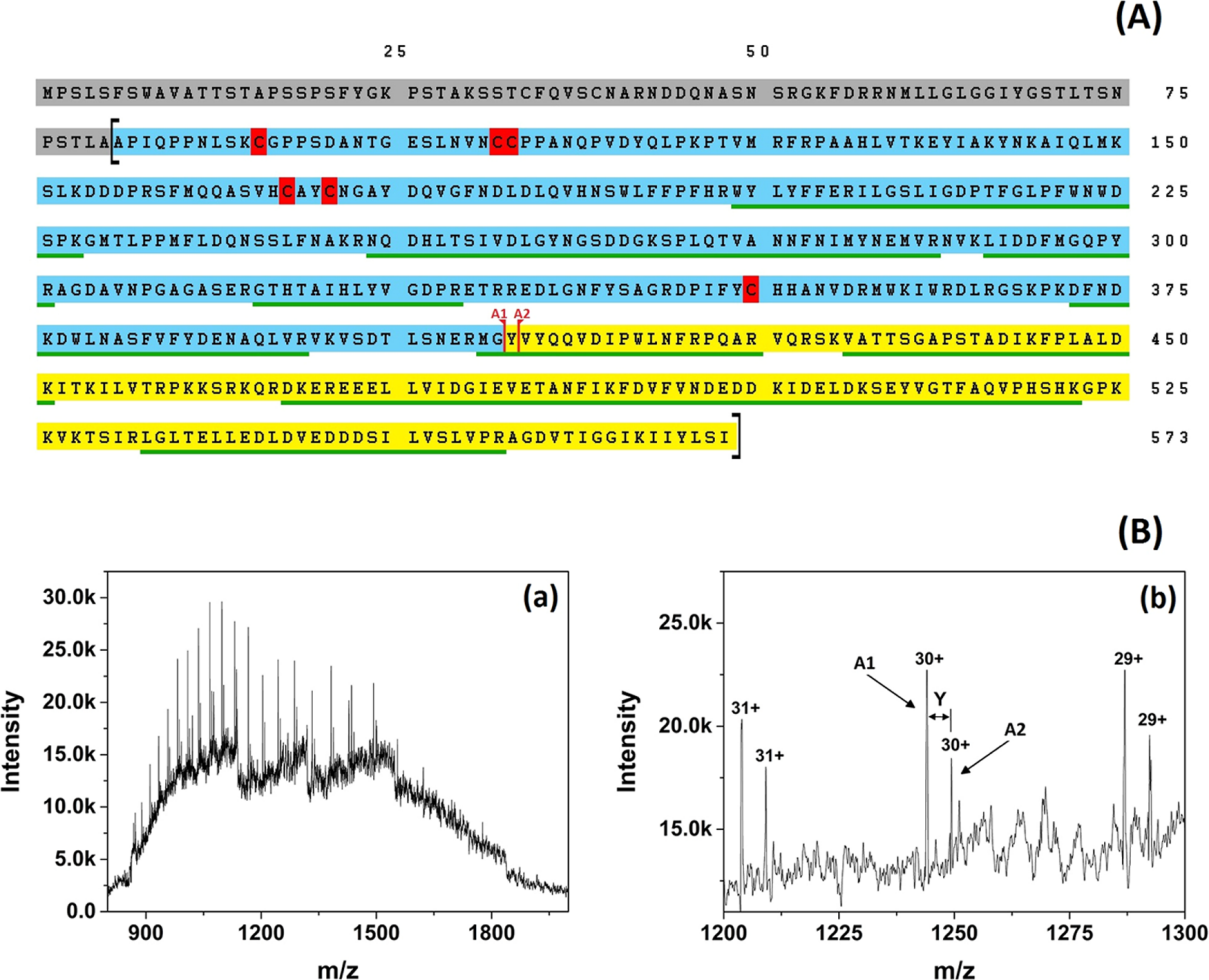
Sequence confirmation and mass determination of *Oe*PPO. (A) Sequence of *Oe*PPO1. Gray shading: signal peptides. Cyan shading: main domain (Active *Oe*PPO1). Yellow shading: C-terminal domain. Red shading: cysteine residues of the main domain. Square brackets indicate the start and the end of Latent *Oe*PPO. Red vertical lines indicate the two cleavage positions (A1 and A2) (the end of Active *Oe*PPO and the start of the C-terminal domain) as deduced from matching the amino acid sequence with the molecular mass determined for *Oe*PPO1 by MS. The peptides identified by UHPLC-ESI-MS/MS for *Oe*PPO1 (INSDC OY733096) are underlined in green. (B) Mass spectra of the purified *Oe*PPO1. (a) The full mass spectrum of *Oe*PPO. The charge state distributions (ranging from about 22 to up to more than 42 charges) indicate the presence of two major protein species (A1 and A2). (b) Zoomed section of charge states [*Oe*PPO + 31H]^31+^ to [*Oe*PPO + 29H]^29+^. The average molecular masses for the species A1 = 37,290.78 ± 0.331 Da (Ala^81^ → Gly^407^) and A2 = 37,453.41 ± 0.387 Da (Ala^81^ → Tyr^408^).

The sequence identification of the purified *Oe*PPO using tryptic digestion followed by UHPLC-ESI-MS/MS unambiguously affirmed the purified enzyme’s identity as olive *Oe*PPO1 (INSDC entry OY733096) with a sequence coverage of 48.2% across the mature protein and the identification of 14 unique peptides covering both domains (main and C-terminal). The identified unique peptides are underlined in green in Figure 6 and listed in Table S3.

The mass spectra of the purified *Oe*PPO1 enzyme indicated the presence of two major protein species (A1 and A2). Twenty distinct peaks were utilized for deconvoluting the charge state distribution of the most significant species A1, and 17 peaks were utilized for species A2. The two species demonstrated deconvoluted masses of 37,290.78 ± 0.331 and 37,453.41 ± 0.387 Da, respectively. These masses precisely corresponded to the theoretical masses of the polypeptides Ala81 → Gly407 (37,292.595 Da) and Ala81 → Tyr408 (37,455.768 Da) of *Oe*PPO1, respectively. This alignment was accomplished by considering either a closed disulfide bridge or a thioether bridge (−2.016 Da), encompassing two cysteines among the six cysteine residues inherent to the peptide chain of the primary domain. These findings corroborate the outcomes of protein identification attained through tryptic digestion, compellingly establishing the purified *Oe*PPO as *Oe*PPO1.

These results suggest that *Oe*PPO1 is likely the only variant expressed within the fruit among the cloned *Oe*PPO genes identified in the fruits or that the other variants are expressed significantly less compared to *Oe*PPO1. Similar observations were reported by Sánchez et al.,^11^ where only two *Oe*PPO genes were found to be significantly expressed compared to other *Oe*PPO genes, and they reported *Oe*PPO1 as the most highly expressed *Oe*PPO gene. Notably, the *Oe*PPO1 (INSDC OY733096) identified herein is 98.8% identical to *Oe*PPO1 (GenBank MW038828) identified by Sánchez et al.^11^

The mass of the latent *Oe*PPO1 form at 54 kDa was not detected by mass spectrometry, suggesting that the intact enzyme did not withstand the mass experiment and underwent dissociation and cleavage during the analysis. The observed masses strongly indicate cleavage events occurring after Gly407 and Tyr408. Furthermore, the mass difference of 162.6 Da between the two species aligns with the loss of the Tyr408 residue (163.17 Da). The measured masses harmoniously fall within the mass range characterizing the active PPO form. Moreover, the two cleavage sites reside within the linker loop located between the tyrosinase domain and the C-terminal domain, preserving the structural integrity of the main tyrosinase domain (Figure S2). This incontrovertibly establishes that the two species (A1 and A2) are active *Oe*PPO1 forms, cleaved at distinct positions. Prior reports^26,36^ have indicated the existence of multiple cleavage positions, suggesting that the removal of the enzyme’s C-terminal lacks a unique cleavage site but encompasses a specific region.

In addition to the two prominent active form species, a number of minor peaks were identified via mass spectrometry as peptide fragments originating from the C-terminal domain (Table S4). These peptides, numbering more than 10, are localized postcleavage sites A1/A2 (Figure 6A). These fragments cover the entire expanse of the C-terminal domain, encompassing three primary initiating sequences (Tyr408, Val409, and Tyr410). The vulnerability of the C-terminal attachment to the main core through the linker loop is well documented in PPOs, where it can be either removed or displaced by various factors such as acids, detergents, and heat.^4^ This fragility serves as a mechanism that facilitates the easier detachment of the C-terminal segment, granting access to the active site and, subsequently, activating the enzyme. However, the strength of the attachment of the C-terminal domain to the main core varies among the different PPOs. While some latent PPOs^37^ can withstand denaturing conditions (such as heat and reducing agents), others^14^ fail to endure reducing conditions due to the C-terminal domain being linked to the main core through disulfide bridges that break in the presence of reducing agents like β-mercaptoethanol. Moreover, in certain PPOs, the C-terminal domain can be cleaved spontaneously during storage.^36^

In the present study, the C-terminal domain of *Oe*PPO1 lacks cysteine residues, ruling out the possibility of forming disulfide bonds that would reinforce its attachment to the main enzyme core (this was confirmed through SDS-PAGE; see Figure 2). Furthermore, the enzyme did not exhibit activation during a three-month storage period (data not shown). However, the enzyme’s structure appears to be highly influenced by heat treatment, as previously evidenced in SDS-PAGE and heat stability analyses. Additionally, it appears that *Oe*PPO is sensitive to mass spectrometry analysis. Nanospray ionization mass spectrometry conditions, such as vaporization and ionization, are known to be gentle on proteins compared with other mass spectrometry techniques. However, the latent form did not withstand these conditions; a similar impact to that observed during heat treatment was observed, resulting in the cleavage or dissociation of the C-terminal domain/subunit. Comparable findings have been documented for oligomeric enzymes during ESI-MS, where the predominant detection is of monomeric subunits rather than intact oligomeric proteins.^59^

The results of the mass spectrometry analysis reinforce the earlier conclusion that the C-terminal part is not covalently bound to the main domain, suggesting that the latent form is oligomeric, composed of two subunits: the main domain subunit and the C-terminal domain subunit. These two subunits are presumably bound together by noncovalent interactions, which were disrupted during heat treatment and ESI-MS conditions.

The assembly of the obtained results suggests that we are witnessing a relatively novel concept of latent PPO activation, where the C-terminal domains can be cleaved or dissociated from the main domain due to high-temperature treatment. This thermal cleavage ultimately yields the denatured active form of *Oe*PPO1.

## Supporting Information Available

Chemical structures for the tested substrates; sequences of cloned/identified *Oe*PPO variants; fluorescence curves of the thermal shift assay; Michaelis–Menten diagrams for the tested substrates; list of primers used; INSDC accession numbers of the cloned/identified *Oe*PPO variants; list of identified peptide sequences of *Oe*PPO1; and peptide fragments identified by mass spectrometry in the C-terminal domain of *Oe*PPO1 (PDF)

## Abbreviations

AEX: anion exchange
ATPS: aqueous two-phase separation
CEX: cation exchange
EDTA: ethylenediaminetetraacetic acid
FPLC: fast protein liquid chromatography
IC50: half-maximal inhibitory concentration
*Oe*PPO: polyphenol oxidase from *Olea europaea*
PEG: poly(ethylene glycol)
PMSF: phenylmethylsulfonyl fluoride
PVP: polyvinylpyrrolidone
PVPP: polyvinylpolypyrrolidone
SDS: sodium lauryl sulfate
SDS-PAGE: sodium dodecyl sulfate-polyacrylamide gel electrophoresis
Tris: tris(hydroxymethyl)aminomethane
UV: ultraviolet
