# Supplementary material for: Extraction, Purification, and Characterization of Olive (Olea europaea L., cv. Chemlal) Polyphenol Oxidase
Source: J Agric Food Chem. 2024 Jan 30;72(6):3099–112. doi: 10.1021/acs.jafc.3c07776 (PMC10870767; doi:10.1021/acs.jafc.3c07776)
Supplement: Supplementary file 1 — jf3c07776_si_001.pdf [file jf3c07776_si_001.pdf]

Supporting information to the article

Extraction, purification and characterization of olive (*Olea europaea* L., cv. Chemlal) polyphenol oxidase

Ala eddine Derardja,[1,2] Matthias Pretzler,[1] Malika Barkat[2] and Annette Rompel[1,*]

[1]Universität Wien, Fakultät für Chemie, Institut für Biophysikalische Chemie, Josef-Holaubek-Platz 2, 1090 Wien, Austria; www.bpc.univie.ac.at.
[2]Laboratoire Bioqual, INATAA, Université des Frères Mentouri, Constantine 1, Route de Ain El-Bey, 25000 Constantine, Algeria.

* FAX: +43-1-4277-852502; Tel.: +43-1-4277-52502; *E-mail: annette.rompel@univie.ac.at.

Summary Information:

Total pages: 9

Total Figures: 4

Total Tables: 4

Table of contents:

Supplementary Figures...S2

Figure S1. Chemical structures for the tested substrates.......................................S2

Figure S2. Comparative alignment of *Oe*PPO sequences.......................................S3

Figure S3. Fluorescence curves of the thermal shift assay of *Oe*PPO1.......................S4

Figure S4. Michaelis-Menten diagrams and chemical structures for the tested substrates....S5

Supplementary Figures...S6

Table S1. List of primers used in this study...S6

Table S2. INSDC Accession numbers of the cloned/identified *Oe*PPO variants..................S6

Table S3. List of identified peptide sequences of *Oe*PPO1 (Ala81 → Ile573)......................S8

Table S4. Peptide fragments identified by Mass Spectrometry (LC/ESI-MS) in the C-Terminal Domain of *Oe*PPO1 (Tyr410 → Ile573)..S9

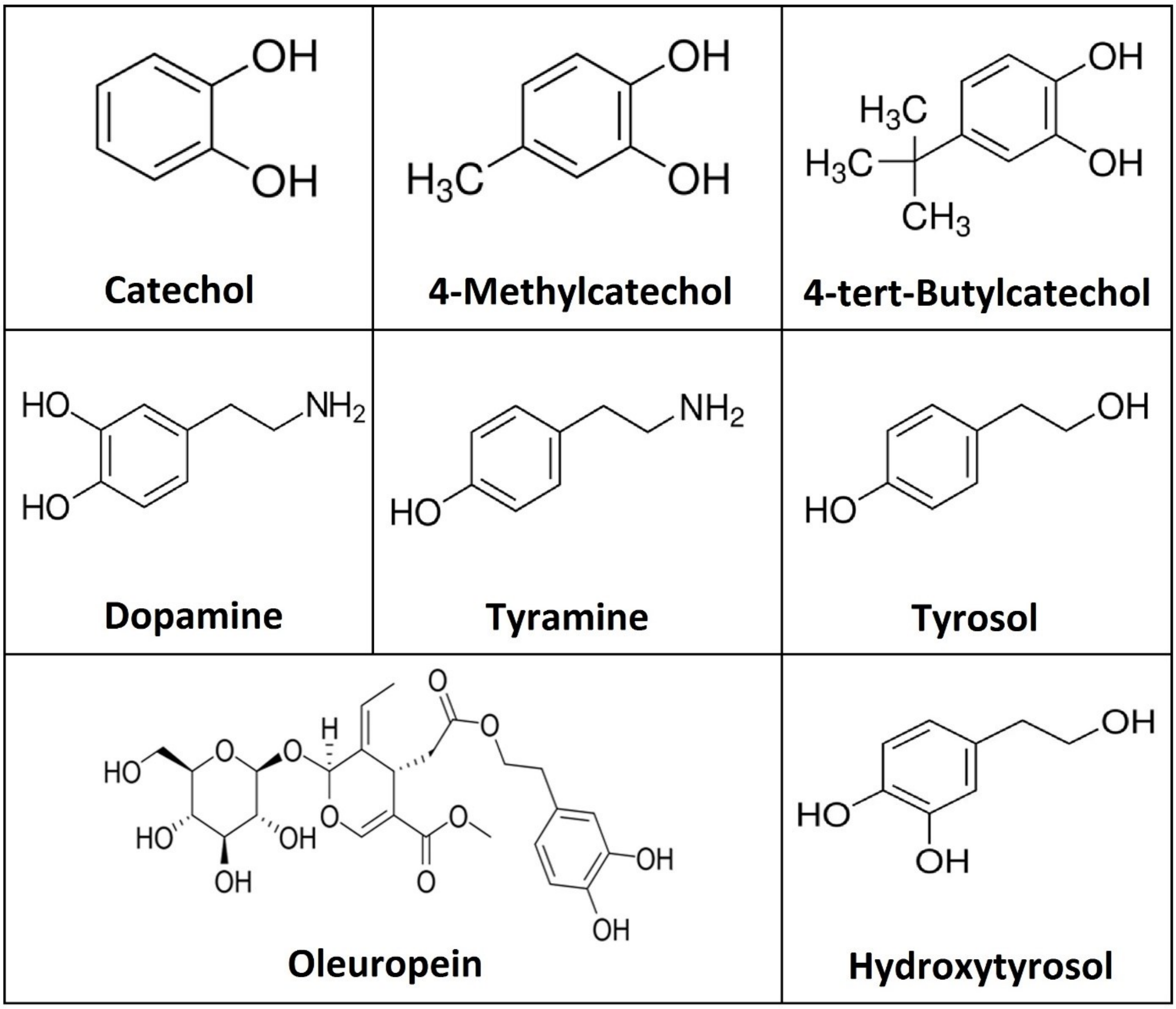

Figure S1. Chemical structures for the tested substrates. Diphenols (catechol, 4-methylcatechol, 4-tert-butylcatechol, dopamine, oleuropein and hydroxytyrosol). Monophenols (tyramine and tyrosol).

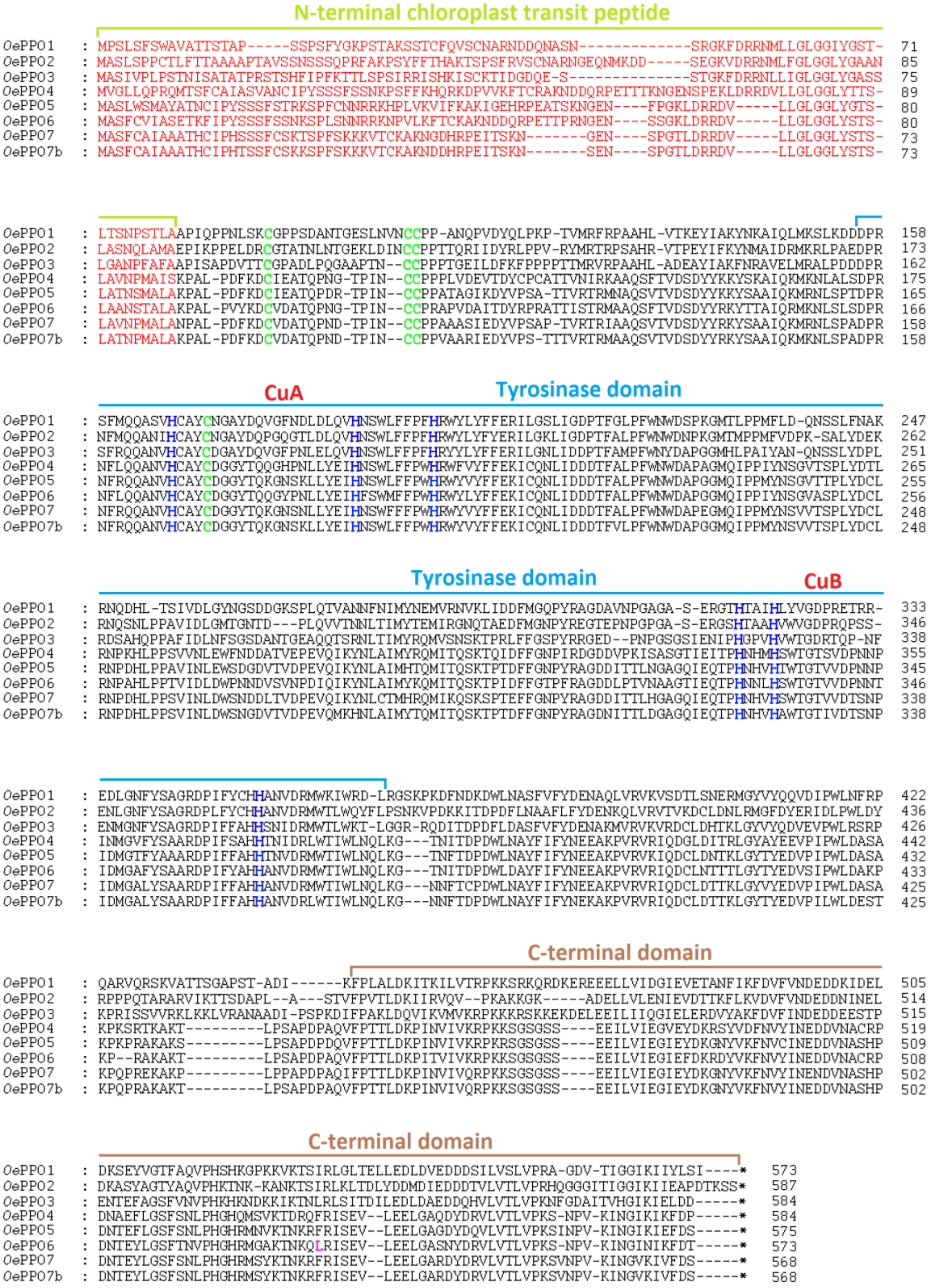

Figure S2. Comparative alignment of *Oe*PPO sequences. Cloned *Oe*PPO sequences (*Oe*PPO1, *Oe*PPO3, *Oe*PPO5, *Oe*PPO6, *Oe*PPO7 and *Oe*PPO7b). *Oe*PPO sequences identified by Sánchez *et al.*[11] (*Oe*PPO2, *Oe*PPO4). *Oe*PPO1 and *Oe*PPO3 were also identified by Sánchez *et al.*[11]. **In red**, signal peptide domain; **In black**, pro-enzyme; **In blue**, copper coordinating histidines; **In pink**, mutation site (L534 →**F**534 in *Oe*PPO6); **In green**, conserved cysteines involved in the disulfide bonds; Green line, indicates the N-terminal chloroplast transit peptide region; blue line, indicates the tyrosinase domain; brown line, indicates the *C*-terminal domain.

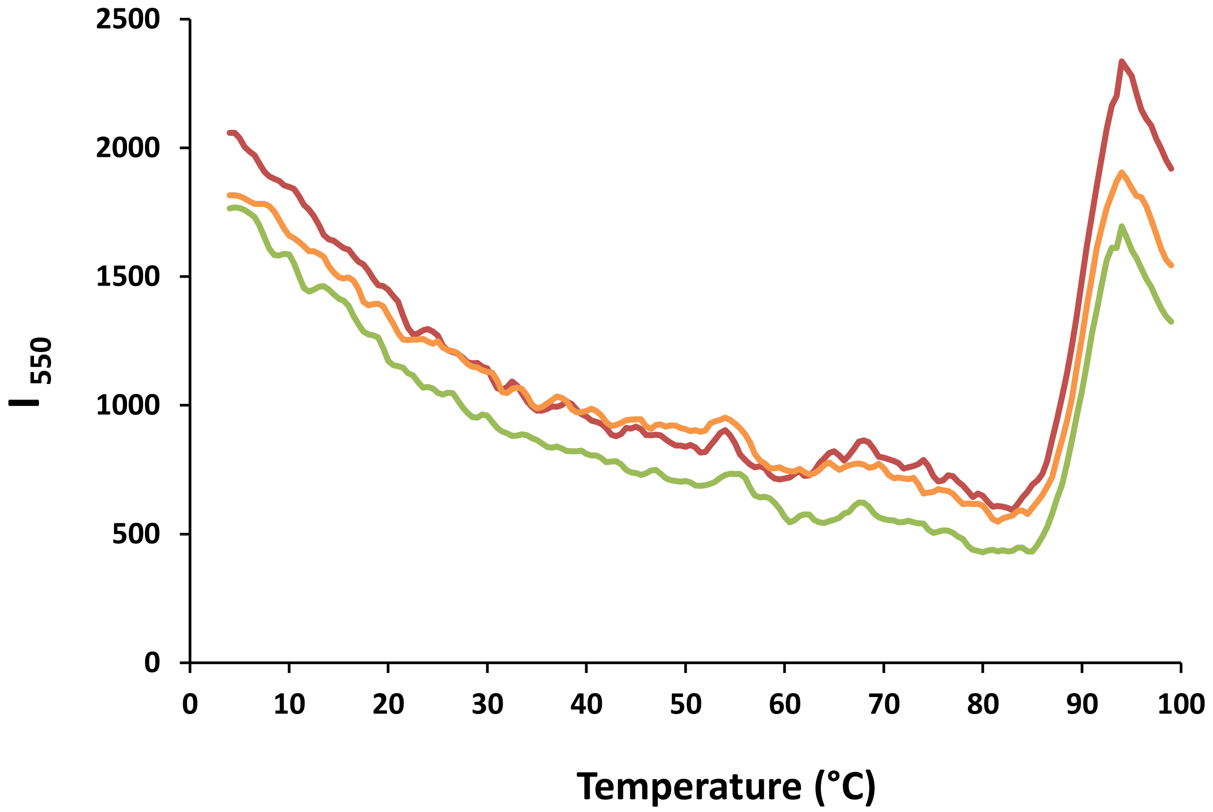

Figure S3. Fluorescence curves of the thermal shift assay of *Oe*PPO1.
I, fluorescence intensity at 550 nm (arbitrary units). The three curves represent measurements of *Oe*PPO1 fluorescence.

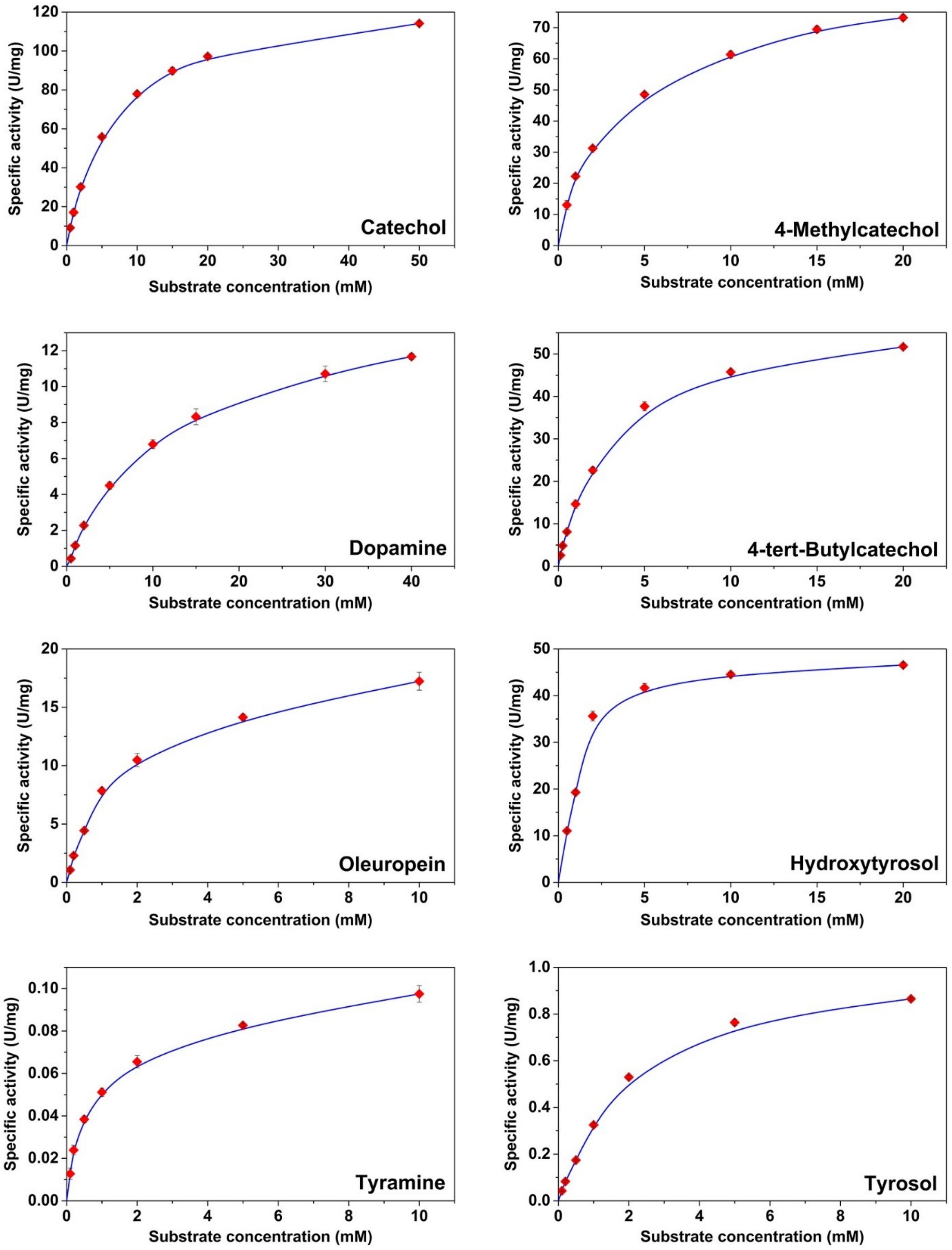

Figure S4. Michaelis-Menten diagrams for the tested substrates.

Red squares represent the average of three measured slopes. Error bars represent the standard deviation, which has been estimated based on data obtained from three replicates.

Table S1. List of primers used in this study.

Name	Sequence (5' to 3')	Target
1539	AGCGGCTCTTCAATGCACAGCATCGATTCACTAAATAAAAGACAATTGCC	*Oe*PPO1
1540	AGCGGCTCTTCTCCCGTACACTCTGCTGACTTCTTAAGTAGAAAATAAATGAGTTC	
1541	AGCGGCTCTTCAATGCCTCTATAAATGATGATCCAAGTAACTTCGTAATATTTAAC TTCAAAGAAAG	*Oe*PPO3
1542	AGCGGCTCTTCTCCCGAAAGGCAGTACACGAAATGAAAGTTGATCA	
1543	AGCGGCTCTTCAATGCTGGTGGTACTTCTGCAAACTAGACAG	*Oe*PPOX
1544	AGCGGCTCTTCTCCCCATAAAACACTTACGAGGATATCTTATTGATAATTCATAAT TATGCCAAATACC	
1545	AGCGGCTCTTCAATGGAATGTCTTGGTACTTCTGCAAACTAGACAG	*Oe*PPOY
1546	AGCGGCTCTTCTCCCTCATGATTATGCCAAGTATTATCTTTTGCAGCAATTTG	
1547	AGCGGCTCTTCAATGGGTGGTACTTCTGCAAACTAGACGTAG	*Oe*PPOZ
1548	AGCGGCTCTTCTCCCGACTGCTACGAAATATTTTGCGGCAATTTG	

Table S2. INSDC Accession numbers of the cloned/identified *Oe*PPO variants.

INSDC Accession number	Gene name
OY733096	*oe*PPO1
OY733097	*oe*PPO3
OY733098	*oe*PPO5
OY733099	*oe*PPO6
OY733100	*oe*PPO6
OY733101	*oe*PPO7
OY733102	*oe*PPO7b

>Intron in OY733097
```
Gtaacgttgaactttctaatctaaaagcttattcatcataatcgaggtcaacatctttgttgacttagaccatc
tcttatgtaaaatagttaaattgcccaattttatttacttaaattttgtcttggacag
```

```
>OY733096_OePPO1-Chemlal
MPSLSFSWAVATTSTAPSSPSFYGKPSTAKSSTCFQVSCNARNDDQNASNSRGKFDRRNMLLGLGGIYGSTLTS
NPSTLAAPIQPPNLSKCGPPSDANTGESLNVNCCPPANQPVDYQLPKPTVMRFRPAAHLVTKEYIAKYNKAIQL
MKSLKDDDPRSFMQQASVHCAYCNGAYDQVGFNDLDLQVHNSWLFFPFHRWYLYFFERILGSLIGDPTFGLPFW
NWDSPKGMTLPPMFLDQNSSLFNAKRNQDHLTSIVDLGYNGSDDGKSPLQTVANNFNIMYNEMVRNVKLIDDFM
GQPYRAGDAVNPGAGASERGTHTAIHLYVGDPRETRREDLGNFYSAGRDPIFYCHHANVDRMWKIWRDLRGSKP
KDFNDKDWLNASFVFYDENAQLVRVKVSDTLSNERMGYVYQQVDIPWLNFRPQARVQRSKVATTSGAPSTADIK
FPLALDKITKILVTRPKKSRKQRDKEREEELLVIDGIEVETANFIKFDVFVNDEDDKIDELDKSEYVGTFAQVP
HSHKGPKKVKTSIRLGLTELLEDLDVEDDDSILVSLVPRAGDVTIGGIKIIYLSI*
```

>OY733097_OePPO3-Chemlal
MASIVPLPSTNISATATPRSTSHFIPFKTTLSPSIRRISHKISCKTIDGDQESSTGKFDRRNLLIGLGGLYGAS
SLGANPFAFAAPISAPDVTTCGPADLPQGAAPTNCCPPPTGEILDFKFPPPPTTMRVRPAAHLADEAYIAKFNR
AVELMRALPDDDPRSFRQQANVHCAYCDGAYDQVGFPNLELQVHNSWLFFPFHRYYLYFFERILGNLIDDPTFA
MPFWNYDAPGGMHLPAIYANQNSSLYDPLRDSAHQPPAFIDLNFSGSDANTGEAQQTSRNLTIMYRQMVSNSKT
PRLFFGSPYRRGEDPNPGSGSIENIPHGPVHVWTGDRTQPNFENMGNFYSAGRDPIFFAHHSNIDRMWTLWKTL
GGRRQDITDPDFLDASFVFYDENAKMVRVKVRDCLDHTKLGYVYQDVEVPWLRSRPKPRISSVVRKLKKLVRAN
AADIPSPKDIFPAKLDQVIKVMVKRPKKKRSKKEKDELEEILIIQGIELERDVYAKFDVFINDEDDEESTPENT
EFAGSFVNVPHKHKNDKKIKTNLRLSITDILEDLDAEDDQHVLVTLVPKNFGDAITVHGIKIELDD*

>OY733098_OePPO5-Chemlal
MASLWSMAYATNCIPYSSSFSTRKSPFCNNRRKHPLVKVIFKAKIGEHRPEATSKNGENFPGKLDRRDVLLGLG
GLYGTSLATNSMALAKPALPDFKDCIEATQPDRTPINCCPPATAGIKDYVPSATTVRTRMNAQSVTVDSDYYKK
YSAAIQKMRNLSPTDPRNFRQQANVHCAYCDGGYTQKGNSKLLYEIHNSWLFFPWHRWYVYFFEKICQNLIDDD
TFALPFWNWDAPGGMQIPPMYNSGVTTPLYDCLRNPDHLPPAVINLEWSDGDVTVDPEVQIKYNLAIMHTQMIT
QSKTPTDFFGNPYRAGDDITTLNGAGQIEQTPHNHVHTWTGTVVDPNNPIDMGTFYAAARDPIFFAHHTNVDRM
WTIWLNQLKGTNFTDPDWLNAYFIFYNEEAKPVRVKIQDCLDNTKLGYTYEDVPIPWLDASAKPKPRAKAKSLP
SAPDPDQVFPTTLDKPINVIVKRPKRSGSGSSEEILVIEGIEYDKGNYVKFNVCINEDDVNASHPDNTEFLGSF
SNLPHGHRMNVKTNKRFRISEVLEELGAGDYDQVLVTLVPKSINPVKINGIKIEFDS*

>OY733099_OePPO6-Chemlal
MASFCVIASETKFIPYSSSFSSNKSPLSNNRRKNPVLKFTCKAKNDDQRPETTPRNGENSSGKLDRRDVLLGLG
GLYSTSLAANSTALAKPALPVYKDCVDATQPNGTPINCCPRAPVDAITDYRPRATTISTRMAAQSFTVDSDYYR
KYTTAIQRMKNLSLSDPRNFLQQANVHCAYCDGGYTQQGYPNLLYEIHFSWMFFPWHRWYLYFFEKICQNLIDD
DTFALPFWNWDAPGGMQIPPIYNSGVASPLYDCLRNPAHLPPTVIDLDWPNNDVSVNPDIQIKYNLAIMYKQMI
TQSKTPIDFFGTPFRAGDDLPTVNAAGTIEQTPHNNLHSWTGTVVDPNNTIDMGAFYSAARDPIFYAHHANVDR
MWTIWLNQLKGTNITDPDWLDAYFIFYNEEAKPVRVRIQDCLNTTTLGYTYEDVSIPWLDAKPKPRAKAKTLPS
APDPAQVFPTTLDKPITVIVKRPKKSGSGSSEEILVIEGIEFDKRDYVKFNVYINEDDVNACRPDNTEYLGSFT
NVPHGHRMGAKTNKQLRISEVLEELGASNYDRVLVTLVPKSNPVKINGINIKFDT*

>OY733100_OePPO6-Chemlal
MASFCVIASETKFIPYSSSFSSNKSPLSNNRRKNPVLKFTCKAKNDDQRPETTPRNGENSSGKLDRRDVLLGLG
GLYSTSLAANSTALAKPALPVYKDCVDATQPNGTPINCCPRAPVDAITDYRPRATTISTRMAAQSFTVDSDYYR
KYTTAIQRMKNLSLSDPRNFLQQANVHCAYCDGGYTQQGYPNLLYEIHFSWMFFPWHRWYLYFFEKICQNLIDD
DTFALPFWNWDAPGGMQIPPIYNSGVASPLYDCLRNPAHLPPTVIDLDWPNNDVSVNPDIQIKYNLAIMYKQMI
TQSKTPIDFFGTPFRAGDDLPTVNAAGTIEQTPHNNLHSWTGTVVDPNNTIDMGAFYSAARDPIFYAHHANVDR
MWTIWLNQLKGTNITDPDWLDAYFIFYNEEAKPVRVRIQDCLNTTTLGYTYEDVSIPWLDAKPKPRAKAKTLPS
APDPAQVFPTTLDKPITVIVKRPKKSGSGSSEEILVIEGIEFDKRDYVKFNVYINEDDVNACRPDNTEYLGSFT
NVPHGHRMGAKTNKQFRISEVLEELGASNYDRVLVTLVPKSNPVKINGINIKFDT*

>OY733101_OePPO7-Chemlal
MASFCAIAAATHCIPHSSSFCSKTSPFSKKKVTCKAKNGDHRPEITSKNGENSPGTLDRRDVLLGLGGLYSTSL
AVNPMALANPALPDFKDCVDATQPNDTPINCCPPAAASIEDYVPSAPTVRTRIAAQSVTVDSDYYRKYSAAIQK
MRNLSPADPRNFRQQANVHCAYCDGGYTQKGNSNLLYEIHNSWFFFPWHRWYVYFFEKICQNLIDDDTFALPFW
NWDAPEGMQIPPMYNSVVTSPLYDCLRNPDHLPPSVINLDWSNDDLTVDPEVQIKYNLCTMHRQMIKQSKSPTE
FFGNPYRAGDDITTLHGAGQIEQTPHNHVHSWTGTVVDTSNPIDMGALYSAARDPIFFAHHANVDRMWTIWLNQ
LKGNNFTCPDWLNAYFIFYNEEAKPVRVRIQDCLDTTKLGYVYEDVPIPWLDASAKPQPREKAKPLPPAPDPAQ
IFPTTLDKPINVIVQRPKKSGSGSSEEILVIEGIEYDKGNYVKFNVYINENDVNASHPDNTEYLGSFSNLPHGH
RMSYKTNKRFRISEVLEELGARDYDRVLVTLVPKSVNPVKINGVKIVFDS*

>OY733102_OePPO7b-Chemlal
MASFCAIAAATHCIPHTSSFCSKKSPFSKKKVTCKAKNDDHRPEITSKNSENSPGTLDRRDVLLGLGGLYSTSL
ATNPMALAKPALPDFKDCVDATQPNDTPINCCPPVAARIEDYVPSTTTVRTRMAAQSVTVDSDYYRKYSAAIQK
MKNLSPADPRNFRQQANVHCAYCDGGYTQKGNSKLLYEIHNSWLFFPWHRWYVYFFEKICQNLIDDDTFVLPFW
NWDAPGGMQIPPMYNSVVTSPLYDCLRNPDHLPPSVINLDWSNGDVTVDPEVQMKHNLAIMYTQMITQSKTPTD
FFGNPYRAGDNITTLDGAGQIEQTPHNHVHAWTGTIVDTSNPIDMGALYSAARDPIFFAHHANVDRLWTIWLNQ
LKGNNFTDPDWLNAYFIFYNEKAKPVRVRIQDCLDTTKLGYTYEDVPILWLDESTKPQPRAKAKTLPSAPDPAQ
VFPTTLDKPINVIVQRPKKSGSGSSEEILVIEGIEYDKGNYVKFNVYINEDDVNASHPDNTEYLGSFSNLPHGH
RMSYKTNKRFRISEVLEELGARDYDRVLVTLVPKSVNPVKINGVKIVFDS*

Table S3. List of identified unique peptide sequences of *Oe*PPO1 (Ala81 → Ile573).

Positions	Peptide Sequences*	Domain
199-206	WYLYFFER	Main Domain
207-228	ILGSLIGDPTFGLPFWNWDSPK	Main Domain
249-268	NQDHLTSIVDLGYNGSDDGK	Main Domain
269-287	SPLQTVANNFNIMYNEMVR	Main Domain
291-301	LIDDFMGQPYR	Main Domain
316-329	GTHTAIHLYVGDPR	Main Domain
372-394	DFNDKDWLNASFVFYDENAQLVR	Main Domain
377-394	DWLNASFVFYDENAQLVR	Main Domain
406-425	MGYVYQQVDIPWLNFRPQAR	Main Domain / *C*-Terminal Domain
431-451	VATTSGAPSTADIKFPLALDK	*C*-Terminal Domain
468-490	DKEREEELLVIDGIEVETANFIK	*C*-Terminal Domain
491-507	FDVFVNDEDDKIDELDK	*C*-Terminal Domain
508-522	SEYVGTFAQVPHSHK	*C*-Terminal Domain
533-557	LGLTELLEDLDVEDDDSILVSLVPR	*C*-Terminal Domain

*Peptide sequences identified by UHPLC-ESI MS/MS.

Table S4. Peptide fragments identified by Mass Spectrometry (LC/ESI-MS) in the *C*-Terminal Domain of *Oe*PPO1 (Tyr410 → Ile573).

Positions	Peptide Sequences	Theoretical mass (Da)	Determined mass (Da)
Y408-T562	YVYQQVDIPWLNFRPQARVQRSKVATTSGAPSTAD IKFPLALDKITKILVTRPKKSRKQRDKEREEELLV IDGIEVETANFIKFDVFVNDEDDKIDELDKSEYVG TFAQVPHSHKGPKKVKTSIRLGLTELLEDLDVEDD DSILVSLVPRAGDVT	17580.72	17580.86 ± 0.65
Y408-I563	YVYQQVDIPWLNFRPQARVQRSKVATTSGAPSTAD IKFPLALDKITKILVTRPKKSRKQRDKEREEELLV IDGIEVETANFIKFDVFVNDEDDKIDELDKSEYVG TFAQVPHSHKGPKKVKTSIRLGLTELLEDLDVEDD DSILVSLVPRAGDVTI	17693.88	17693.56 ± 0.61
Y408-S572	YVYQQVDIPWLNFRPQARVQRSKVATTSGAPSTAD IKFPLALDKITKILVTRPKKSRKQRDKEREEELLV IDGIEVETANFIKFDVFVNDEDDKIDELDKSEYVG TFAQVPHSHKGPKKVKTSIRLGLTELLEDLDVEDD DSILVSLVPRAGDVTIGGIKIIYLS	18639.04	18639.34 ± 1.01
V409-G564	VYQQVDIPWLNFRPQARVQRSKVATTSGAPSTADI KFPLALDKITKILVTRPKKSRKQRDKEREEELLVI DGIEVETANFIKFDVFVNDEDDKIDELDKSEYVGT FAQVPHSHKGPKKVKTSIRLGLTELLEDLDVEDDD SILVSLVPRAGDVTIG	17587.76	17588.31 ± 0.83
V409-I573	VYQQVDIPWLNFRPQARVQRSKVATTSGAPSTADI KFPLALDKITKILVTRPKKSRKQRDKEREEELLVI DGIEVETANFIKFDVFVNDEDDKIDELDKSEYVGT FAQVPHSHKGPKKVKTSIRLGLTELLEDLDVEDDD SILVSLVPRAGDVTIGGIKIIYLSI	18589.02	18589.76 ± 0.51
Y410-G534	YQQVDIPWLNFRPQARVQRSKVATTSGAPSTADIK FPLALDKITKILVTRPKKSRKQRDKEREEELLVID GIEVETANFIKFDVFVNDEDDKIDELDKSEYVGTF AQVPHSHKGPKKVKTSIRLG	14294.13	14294.39 ± 0.66
Y410-L538	YQQVDIPWLNFRPQARVQRSKVATTSGAPSTADIKF PLALDKITKILVTRPKKSRKQRDKEREEELLVIDGI EVETANFIKFDVFVNDEDDKIDELDKSEYVGTFAQV PHSHKGPKKVKTSIRLGLTEL	14750.67	14750.78 ± 0.05
Y410-L539	YQQVDIPWLNFRPQARVQRSKVATTSGAPSTADIK FPLALDKITKILVTRPKKSRKQRDKEREEELLVID GIEVETANFIKFDVFVNDEDDKIDELDKSEYVGTF AQVPHSHKGPKKVKTSIRLGLTELL	14863.82	14863.38 ± 0.47
Y410-I573	YQQVDIPWLNFRPQARVQRSKVATTSGAPSTADIK FPLALDKITKILVTRPKKSRKQRDKEREEELLVID GIEVETANFIKFDVFVNDEDDKIDELDKSEYVGTF AQVPHSHKGPKKVKTSIRLGLTELLEDLDVEDDDS ILVSLVPRAGDVTIGGIKIIYLSI	18489.89	18489.83 ±0.49
